# A biomimetic engineered bone platform for advanced testing of prosthetic implants

**DOI:** 10.1038/s41598-020-78416-w

**Published:** 2020-12-17

**Authors:** Martina Sladkova-Faure, Michael Pujari-Palmer, Caroline Öhman-Mägi, Alejandro López, Hanbin Wang, Håkan Engqvist, Giuseppe Maria de Peppo

**Affiliations:** 1 The New York Stem Cell Foundation Research Institute, 619 West 54th Street, New York, NY 10019 USA; 2 Division of Applied Materials Sciences, Uppsala University, Uppsala, Sweden

**Keywords:** Biological techniques, Biotechnology, Stem cells, Health care, Medical research, Engineering, Materials science

## Abstract

Existing methods for testing prosthetic implants suffer from critical limitations, creating an urgent need for new strategies that facilitate research and development of implants with enhanced osseointegration potential. Herein, we describe a novel, biomimetic, human bone platform for advanced testing of implants in vitro, and demonstrate the scientific validity and predictive value of this approach using an assortment of complementary evaluation methods. We anchored titanium (Ti) and stainless steel (SS) implants into biomimetic scaffolds, seeded with human induced mesenchymal stem cells, to recapitulate the osseointegration process in vitro. We show distinct patterns of gene expression, matrix deposition, and mineralization in response to the two materials, with Ti implants ultimately resulting in stronger integration strength, as seen in other preclinical and clinical studies. Interestingly, RNAseq analysis reveals that the TGF-beta and the FGF2 pathways are overexpressed in response to Ti implants, while the Wnt, BMP, and IGF pathways are overexpressed in response to SS implants. High-resolution imaging shows significantly increased tissue mineralization and calcium deposition at the tissue-implant interface in response to Ti implants, contributing to a twofold increase in pullout strength compared to SS implants. Our technology creates unprecedented research opportunities towards the design of implants and biomaterials that can be personalized, and exhibit enhanced osseointegration potential, with reduced need for animal testing.

## Introduction

Prosthetic implants are routinely used in dentistry and orthopedics to treat edentulous people and patients affected by skeletal defects, with a global market worth more than 50 billion dollars annually^1^. When a prosthetic implant is placed into bone tissue, a series of molecular and cellular events coordinate the formation of a structural and functional bond between the implant and the surrounding tissue—osseointegration^2,3^. In particular, mesenchymal stem cells (MSC) migrate to the implant surface, attach to a fibronectin framework, differentiate into osteoblasts, and produce an organic matrix that eventually mineralizes as woven bone at the tissue-implant interface via a process referred as intramembranous ossification. This process depends upon the implant surface chemistry and topography, as well as the implant design^4^. While many dental and orthopedic prosthetic implants have a 95% success rate, the evaluation and development process for new generation implants is suboptimal, and it is difficult to ensure that a new implant will quickly establish a strong and enduring bond with the surrounding bone leading to better clinical outcomes^5–7^. For example, one of the most common source of implant failure is peri-implantatitis, which can be prevented by modifying the surface of implants^8^. Unfortunately, available methods to test new implants bear limitations and have become inadequate in light of new emerging technologies. Current two-dimensional (2D) cell culture methods fail to depict the cytoarchitecture typical of native bone environment, and do not enable cell-to-cell and cell-to-matrix interactions that are critical to controlling cell fate and tissue functions in vivo^9^. Importantly, as these methods cannot measure the mechanical interaction between the tissue and the implant, they cannot predict the osseointegration potential of new prosthetic implants. The standard evaluation method for testing the mechanical stability of new implants is pullout testing in artificial polyurethane foams (PU)^10^ or human cadaveric bone^11^, and in preclinical animal models^12^. While PU foams and cadaveric bone offer some insight into the primary stability of new implant designs, animal studies are the closest option for recapitulating the biological response to implants, and to help predict the integration efficiency of new implant systems. Unfortunately, animal studies are time and resource intensive, and fundamentally unreliable due to interspecies differences in tissue quality, physiology, and metabolism^13^. Therefore, the overall testing ability and predictive power of existing methods is poor^14^, indicating a need for new, robust, human-relevant systems that strongly correlate with clinical outcomes. By using a biomimetic approach to bone development, we recently engineered functional bone tissue, i.e. displaying architectural and biological features typical of the native tissue, from human induced pluripotent stem cells (iPSC)^15,16^, with significant potential to serve as replacement product for clinical applications and as an in vitro system for advanced screening of drugs and biomaterials.

In this study, we have developed a novel, biomimetic platform to test titanium (Ti) and stainless steel (SS) implants. These implants elicit different biological responses in vivo, which ultimately lead to different integration strengths^17,18^. We have anchored the implants into decellularized bone scaffolds and grown bone tissue from human induced MSCs (iMSC) for seven weeks. While other groups have used decellularized bone as a scaffold for bone engineering applications, this is the first attempt to use tissue-engineered bone as a biomimetic system to test implant biomaterials in vitro. Using a combination of molecular biology techniques, biochemical assays, histological methods, imaging procedures, high-resolution surface characterization and biomechanical testing, we have studied the molecular response to the implants, examined the quality of the tissue-implant interface, and measured the strength of the tissue-implant interaction.

This study demonstrates our testing platform can simulate the tissue-implant interaction process in vitro, offering a powerful system to advance the development of new orthopedic implants and biomaterials with enhanced osseointegration potential.

## Results and discussion

### Engineering the human bone-implant platform

**Figure 1:**
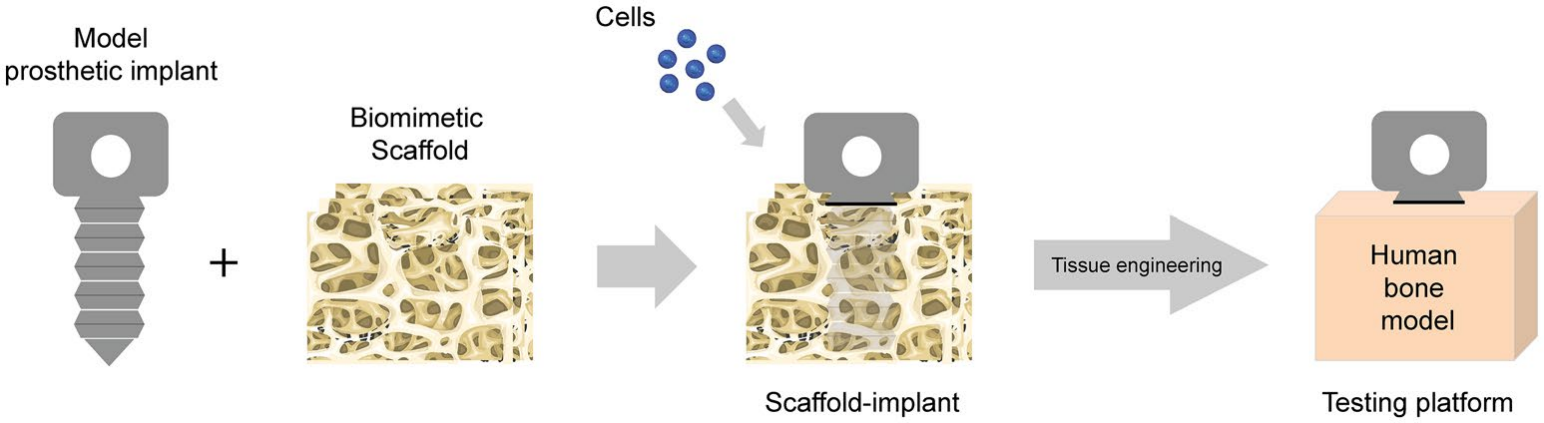
Engineering the bone-implant platform. Model prosthetic implants are anchored to biomimetic scaffolds seeded with bone-forming cells. Following culture in an osteogenic environment, the samples are examined to study the tissue response to the implants, the quality of the tissue-implant interface, and the strength of interaction between the implant and the tissue-engineered bone. Additional results are shown in Figs. S1, S2, S3 and Table S1.

To build a testing platform for advanced characterization of bone implants, we anchored model prosthetic Ti and SS implants into decellularized bone scaffolds, seeded the implant-scaffold constructs with iMSCs, and cultured the samples in an osteogenic environment in the presence of osteogenic inducing factors to grow bone tissue in vitro (Fig. 1, Fig. S1).

We tested Ti and SS implants since these materials produce different outcomes in vivo^17,18^, with Ti implants leading to the formation of a stronger bond with the surrounding bone compared to stainless steel implants. Thus, we chose these materials to assess the predictive value of our testing platform in reproducing aspects of the in vivo response to biomaterials. We used decellularized cow bone scaffolds because their trabecular architecture provides a good representation of the tissue environment at the site of implantation^19^, and because they support osteogenic differentiation of human mesenchymal stem cells equally well as scaffolds derived from human bone^20^. We used human iPSCs because they can be derived from different individuals and represent a single cell source that can give rise to all cell types involved in the osseointegration process^21^, enabling to potentially model, autologously, different aspects of the bone response to implants in vivo.

**Figure 2:**
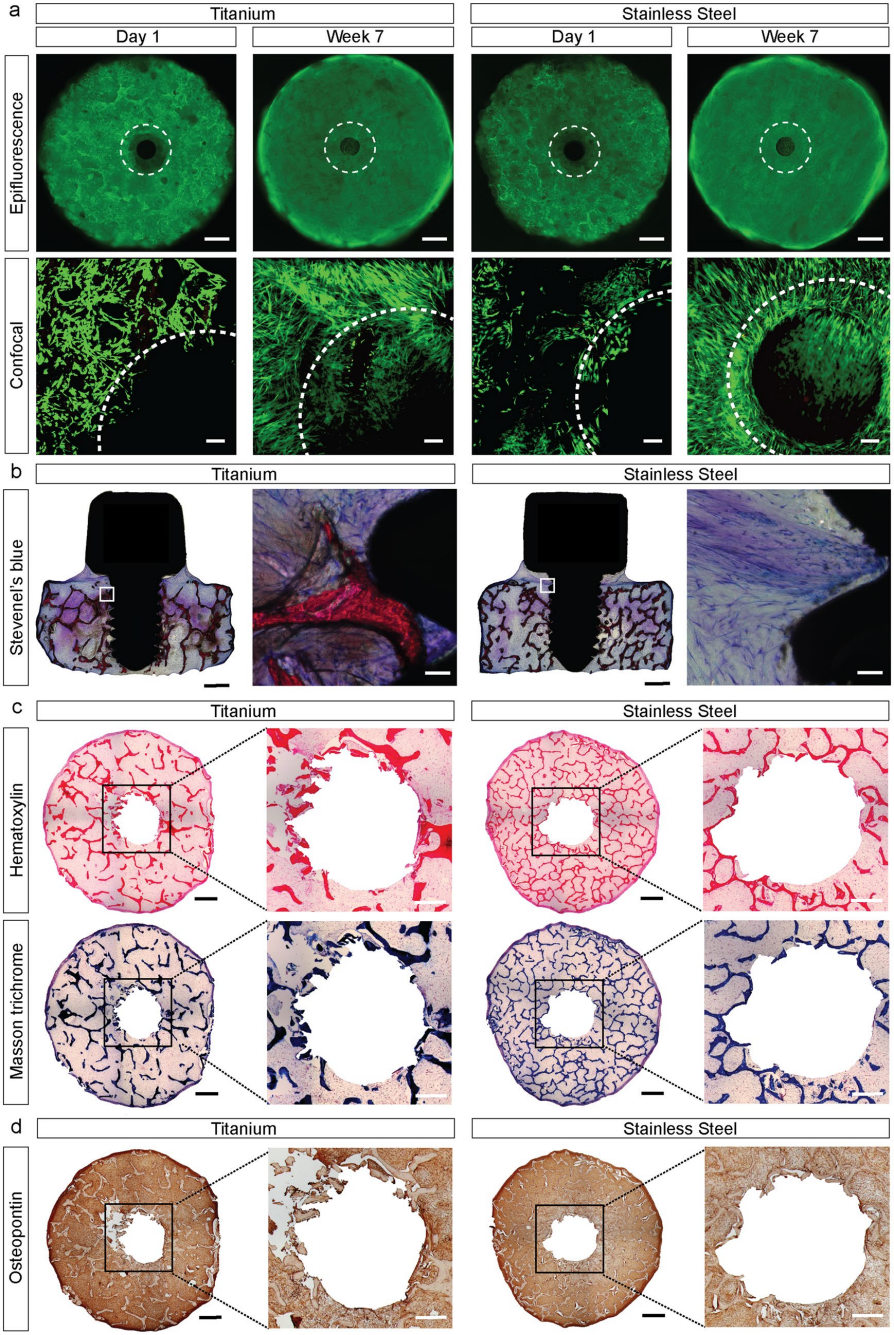
Tissue formation. (**a**) Epifluorescence micrographs (mosaic) and higher magnification confocal images showing distribution of live cells (green) after 1 day and 7 weeks of culture in an osteogenic environment. Dashed lines delimit the boundaries between the tissue and the implants. Scale bar: 1 mm (top) and 100 μm (bottom). (**b**) Histological analysis of non-demineralized resin-embedded samples after 7 weeks of culture in an osteogenic environment. Scale bar: 1 mm (left) and 100 µm (right). (**c**) Histological analysis of demineralized paraffin-embedded samples after 7 weeks of culture in an osteogenic environment. Scale bar: 1 mm (left) and 100 µm (right). (**d**) Immunohistochemical analysis of samples after 7 weeks of culture in an osteogenic environment. Samples are positive (brown) for osteopontin. Scale bar: 1 mm (left) and 100 µm (right). Additional results are shown in Figs. S4, S5 and S6.

In order to minimize the effects of topography and implant design on the tissue response, both Ti and SS implants were manufactured with the same design (Fig. S2a), and with corresponding values of surface roughness (Fig. S2b), as evidenced by SEM imaging and optical profilometry. The trabecular architecture and the number of recruited cells to the site of implantation can affect the tissue response to implants and the strength of any resulting tissue-implant interaction^22^. To reduce the effects of these variables, we selected and matched scaffolds with equivalent density (Table S1) for each analysis, and seeded the cells using an optimized droplet technique, which is highly reproducible (Fig. S3), and results in nearly 100% cell attachment (Fig. S4). Imaging of viable cells and histological assessment confirms cell survival and proliferation (Fig. 2a), and formation of thick tissue (Fig. 2b), throughout the sample interior and on the implant surface, after 7 weeks of culture in an osteogenic environment. The newly formed tissue exhibits typical features of the bone extracellular matrix, characterized by the presence of collagen fibers (Fig. 2c) and the noncollagenous glycoproteins osteopontin, osteocalcin, and bone sialoprotein (Fig. 2d, Figs. S5, S6).

In summary, cytological and histological results confirm our platform replicates fundamental biological and structural features of the native tissue environment, which are critical for advanced testing of implants and biomaterials materials in vitro.

### The human bone-implant platform reveals different molecular responses to Ti and SS implants

**Figure 3:**
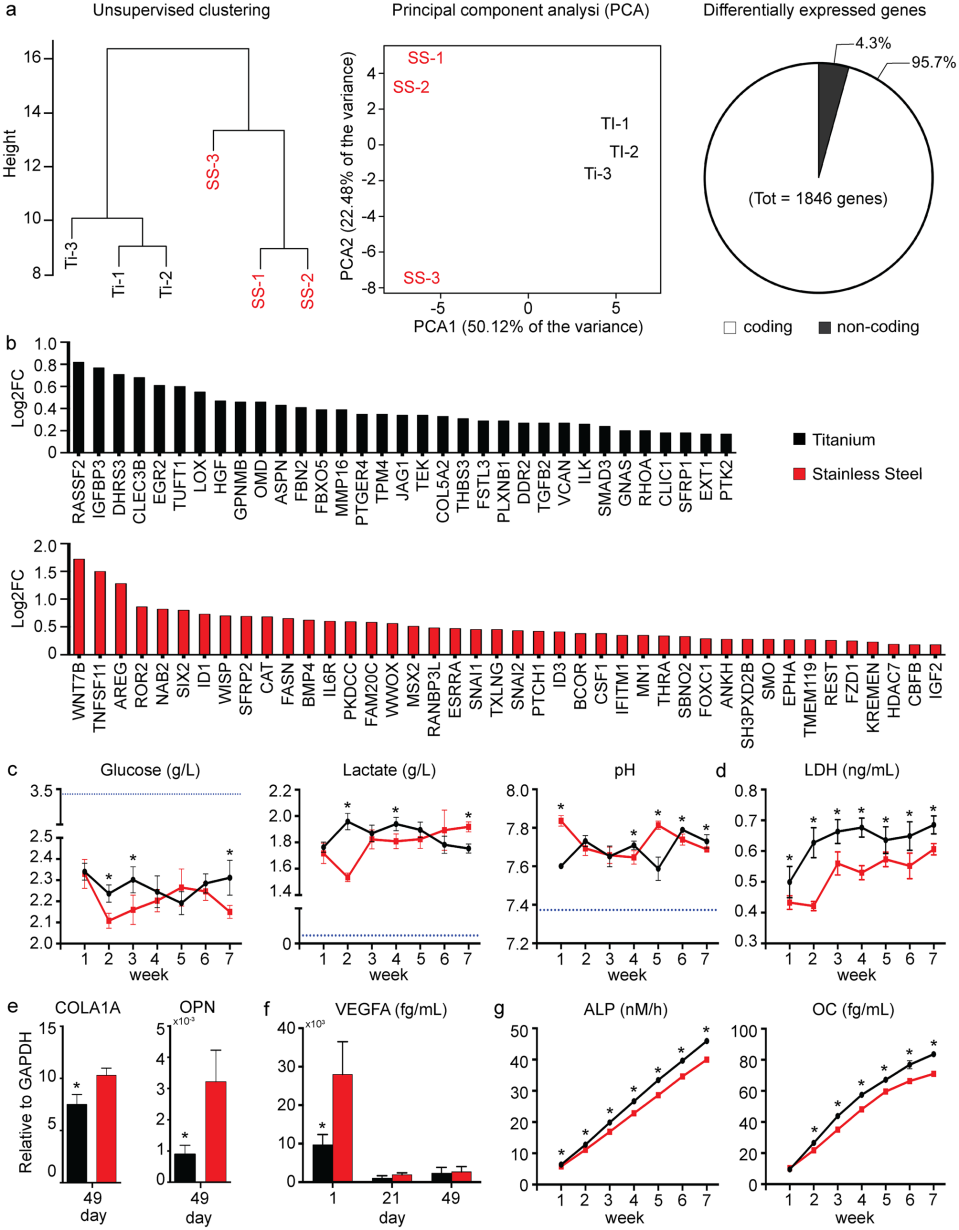
Molecular response to implants. (**a**) RNAseq data showing the clustering analysis, principal component analysis, and number of identified differentially expressed genes between the titanium and stainless steel groups. (**b**) Ossification genes overexpressed in response to titanium and stainless steel implants. (**c**) Profile of culture analytes during tissue growth in response to titanium and stainless steel implants. Data represent averages ± SD (n = 6, unpaired Student’s t-test; asterisk denotes significant difference between the titanium and stainless steel groups). (**d**) Content of lactate dehydrogenase in the medium at the end of each week in an osteogenic environment. Data represent averages ± SD (n = 6, unpaired Student’s t-test; asterisk denotes significant difference between the titanium and stainless steel groups). (**e**) Expression of collagen, type I, alpha 1 (COL1A1) and osteopontin (OPN) at the end of the culture period in response to titanium and stainless steel implants. Data represent averages ± SD (n = 3, unpaired Student’s t-test; asterisk denotes significant difference between the titanium and stainless steel groups). (**f**) Release of vascular endothelial growth factor A (VEGFA) 1 day, 3 weeks and 7 weeks after culture in response to titanium and stainless steel implants. Data represent averages ± SD (n = 3, unpaired Student’s t-test; asterisk denotes significant difference between the titanium and stainless steel groups). (**g**) Cumulative release of alkaline phosphatase (ALP) and osteocalcin (OC) in response to titanium and stainless steel implants. Data represent averages ± SD (n = 6 and 3, unpaired Student t-test; asterisk denotes significant difference between the titanium and stainless steel groups). Additional results are shown in Figs. S7, S8, S9 and S10.

Though the disparate tissue response to Ti and SS implants is well known^17,18^, the molecular mechanisms underlying these differences are poorly understood, in part due to a lack of human specimens. In this study, we used RNAseq analysis to study the global molecular response to Ti and SS implants. Hierarchical clustering and principal component analysis of RNAseq data show the formation of two main groups, with Ti samples clustering together and apart from the SS samples (Fig. 3a). The two materials elicit different responses in human iMSCs, with 1003 genes overexpressed in response to Ti implants (Table S2) and 843 genes overexpressed in response to SS implants (Table S3). Enrichment analysis of differentially expressed genes (DEGs) in GO^23^ returned 729 terms (*p* value < 0.05), including *development*, *cell communication*, *signaling*, *response to stimuli*, *cell proliferation*, and *metabolism* (REVIGO^24^ display in Fig. S7a). Gene enrichment pathway analysis of DEGs (Fig. S7a) returned the *glycolysis/gluconeogenesis* and *cytokine-cytokine receptor interaction* terms (*p* value < 0.05) in KEGG^25^, and the *signal transduction* and *cholesterol biosynthesis* terms (p-value < 0.05) in Reactome^26^. Examination of DEGs with the IPA software^27^ confirms that Ti stimulates the *cholesterol* and *ketogenic biosynthesis* (i.e. linolenate, oleate) pathways, while SS stimulates *glycolytic biosynthesis*, *cytoskeletal signaling*, and *cytokine signaling* (Fig. S7b, Table S4). Regulation of these pathways in response to biomaterials was previously demonstrated^28,29^. For example, cholesterol and products of the cholesterol biosynthesis play a role during osteogenic differentiation of human MSCs^30^ through the formation of lipid rafts^31^ and regulation of the Hedgehog signaling pathway^32^. In this study the lipid raft and caveolae genes CAV1, CAV2, ERLIN2, and PAG1^33^ were overexpressed in response to Ti implants (Table S2), indicating this material induce osteogenic differentiation of human iMSCs through formation of membrane microdomains. Other expression differences are consistent with the known metabolic shift in MSCs, from glycolysis in the proliferative stage, towards oxidative phosphorylation during osteogenic differentiation (Table S2, S3)^34,35^.

Bone development, remodeling and regeneration is controlled by the BMP/TGF-β, Wnt/β-catenin, Notch, Hedgehog, and IGF, and FGF/PTHrP signaling pathways^36,37^. Functional classification of genes involved in the ossification process returned 382 gene products in GO. Of these, 33 genes are overexpressed in response to Ti implants and 42 genes in response to SS implants (Fig. 3b). STRING^38^ analysis reveals the biological functions of these genes are highly connected, with genes overexpressed in response to Ti forming a network composed of 42 edges (enrichment *p* value < 0.001) and genes overexpressed in response to SS forming a network composed of 59 edges (enrichment *p* value < 0.001) (Fig. S8). In particular, implantation of Ti leads to overexpression of canonical and non-canonical TGF-beta pathway components (Fig. S8a and Table S2), perhaps as result of CAV1 and CAV2 expression^39^ (Table S2), while implantation of SS implants leads to overexpression of components of the Wnt, BMP, and IGF signaling pathways (Fig. S8b; Table S3). Notably, cells overexpress fibronectin and fibronectin-related genes in response to Ti implants (Table S2), which are known to enhance the osseointegration potential of both Ti and SS implants in vivo^40,41^. Other important DEGs include the growth factors FGF2, FGF11, CTGF, VEGFA and PDGFC, overexpressed in response to Ti (Table S2), and PDGFA and GDF11, overexpressed in response to SS (Table S3). These factors support cell proliferation but also play a role in differentiation and tissue regeneration^42^. For example, FGF, VEGF, GDF and PDGF are positive regulators of wound healing and bone repair, and can promote/accelerate implant osseointegration in vivo^43–46^.

In agreement with RNAseq data, Ti and SS implants differentially affect the cellular metabolic activity of the cells, and the dynamics of tissue formation. Cells metabolize glucose at a faster rate in response to SS implants, at both early (2–3 weeks, *p* value < 0.001 and 0.003, respectively) and late (7 weeks, *p* value = 0.001) time points, but produce significantly less lactate at 2 weeks (*p* value < 0.001) and 4 weeks (*p* value = 0.001) (Fig. 3c), suggesting that aerobic glycolysis may be preferred in response to Ti implants. Aerobic glycolysis supports cell proliferation^47^ and determines a wide range of cellular functions^48^, including the promotion of osteoblast differentiation and activity^49^. In support of this assumption, Hk2 and Pdk1 genes, which favor lactate over Acetyl-CoA production from pyruvate, are overexpressed in response to Ti implants (Table S2). These findings may also explain the significantly higher content of lactate dehydrogenase (LDH) measured in the medium at all time points (*p* value < 0.005, Table S5) in response to Ti implants (Fig. 3d), perhaps as a result of greater tissue turnover, and the significantly lower medium pH (Fig. 3c) observed at 1 week (*p* value < 0.001) and 5 weeks (*p* value < 0.001) resulting from higher lactate production. Other metabolic enzymes differentially regulated are PFK, PFKC, PFKB4, ALDOA, ALDOC, PGK1, ENO1 and SHMT1, overexpressed in response to Ti implants (Table S2), and MAT2A, overexpressed in response to SS implants (Table S3). In addition to being involved in metabolism, the products of these genes can act as mediators between growth stimuli, signaling pathways, and downstream effectors, and can regulate biological processes such as transcription, proliferation, and apoptosis^50^.

Real-time polymerase chain reaction (qRT-PCR) and enzyme-linked immunosorbent assay (ELISA) further demonstrate that Ti and SS differently affect the expression/production of mesodermal genes and proteins. The expression of COL1A1 (*p* value = 0.01), OPN (*p* value = 0.01), BSP (*p* value = 0.03), and the PPAR-γ (*p* value = 0.01), which has anti-osteoblastogenic effects^51^, at week 7 (Fig. 3e, f, Fig. S9), and the release of VEGFA (*p* value = 0.02) on day 1 (Fig. 3f) are higher in response to SS implants. On the other hand, the release of ALP (*p* value < 0.005, Table S5) and OC (*p* value < 0.01, Table S5) is significantly higher in response to Ti implants during the entire culture period (Fig. 3g).

Altogether, these molecular differences underlie variations in the regeneration process in response to implant materials—in tissue growth rate as well as in synthesis and composition of the extracellular matrix—which are ultimately responsible for the differences in tissue mineralization and tissue-implant interaction strength seen in vivo^17,18^ and reproduced in this study, as discussed in the following sections. These results highlight the advantages of our testing platform, which allow for a deeper understanding of the tissue-scale molecular responses to implants in vitro. Elucidation of the global tissue-implant molecular interactions will aid in the design of new implant systems with better clinical outcomes.

### The human bone-implant platform recapitulates a faster tissue mineralization in response to Ti implants

**Figure 4:**
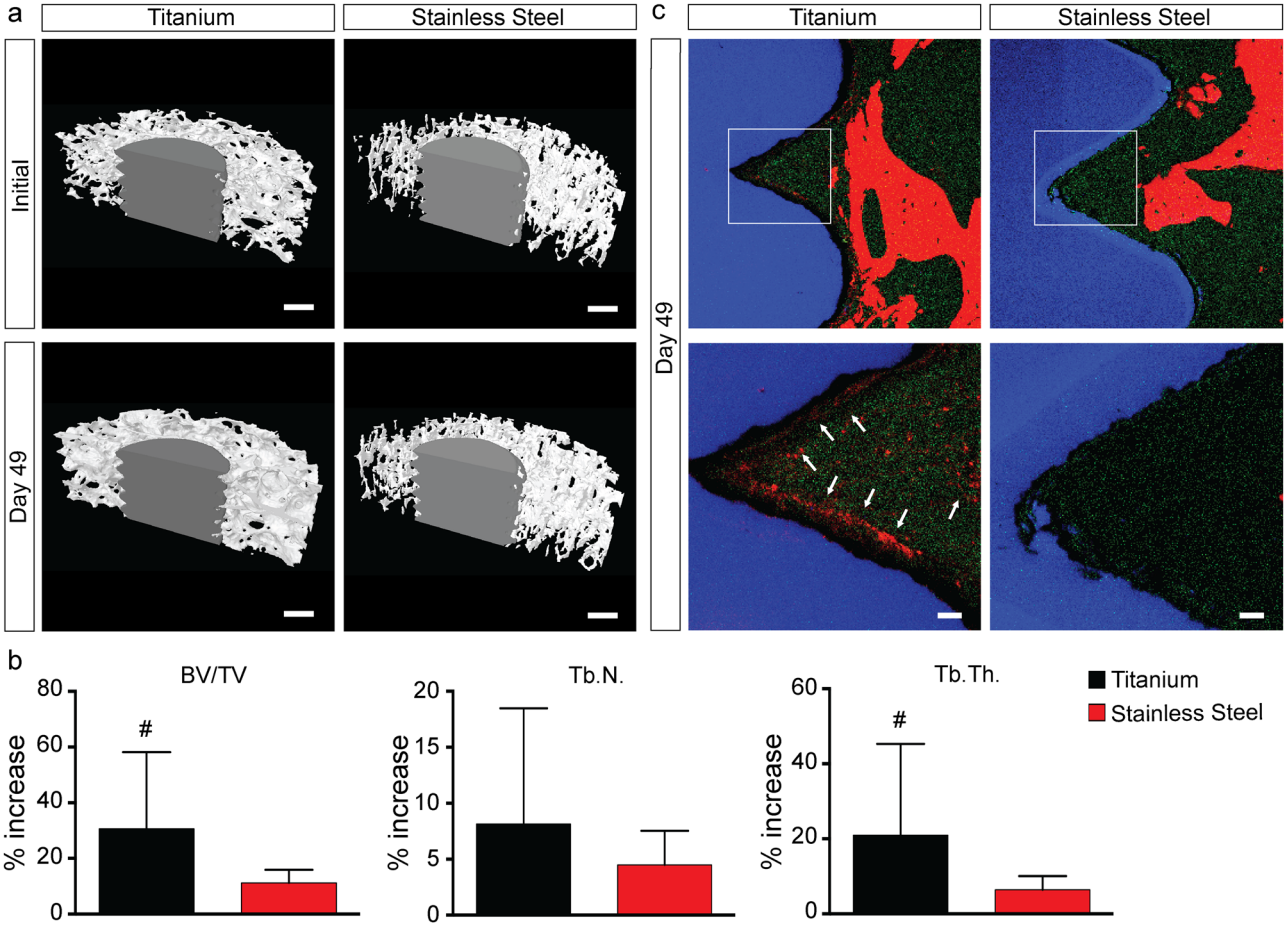
Tissue mineralization. (**a**) Reconstructed three-dimensional microcomputed tomography images of titanium and stainless steel samples before and after 7 weeks of culture in an osteogenic environment. Scale bar: 1 mm. (**b**) Analysis of microcomputed tomography measurements for bone volume over total volume (BV/TV), trabecular number (Tb.N.), and trabecular thickness (Tb.Th.) parameters. Data represent averages ± SD (n = 6, paired and unpaired Student’s t-test; pound denotes significant difference between initial and 49 days). (**c**) Tof–SIMS view of titanium and stainless steel samples after 7 weeks of culture in an osteogenic environments showing the implant (blue), the polymethylmethacrylate (green), and the calcium deposits (red). Scale bar: 50 µm.

After an implant is placed into a bone cavity, MSCs migrate to the implant surface, attach to a fibronectin network, and produce new tissue that eventually mineralizes. Tissue mineralization at the implant surface defines the implant integration process and determines the integration strength. This process is influenced by the surface and bulk physicochemical properties of the implant material as well as by the local tissue environment. In this study, µCT imaging shows that the mineral content significantly increases after 7 weeks of culture in osteogenic conditions for both Ti and SS samples (Fig. 4a). However, Ti implants consistently lead to higher increase in bone structural parameters, with a max percentage increase following culture of 79.16% versus 18.10% for bone volume over total volume (BV/TV), 28.66% versus 8.43% for trabecular number (TB.N.), an 69.72% vs 11.12% for trabecular thickness (Tb.Th.) (Fig. 4b). The higher increase in tissue mineralization correlates with the significantly higher amount of alkaline phosphatase (ALP), a strong predictor of neotissue mineralization^52^, released in response to Ti implants (Table S5). Time-of-flight secondary ion mass spectrometry (Tof–SIMS) confirms that calcium deposits form only on the surface of Ti implants after cell culture in osteogenic conditions (Fig. 4c, Fig. S11), indicating that Ti triggers the formation of a tissue-implant interfacial matrix that mineralizes faster. Our findings corroborate the results seen in other preclinical and clinical studies^16^. Taking into account the ability of this platform to reproduce cellular and molecular events (i.e. tissue formation and ECM production and mineralization) at the implant surface, the present work suggests that it can be used to assess the osteoinductive and osteoconductive properties of new biomaterials in vitro, and effectively predict the osseointegration potential of new implants in vivo.

Meaning our testing platform is suitable to assess the osteoinductive and osteoconductive properties of new implants in vitro, and effectively predict their osseointegration potential in vivo.

### The human bone-implant platform reproduces the integration potential of Ti and SS implants

**Figure 5:**
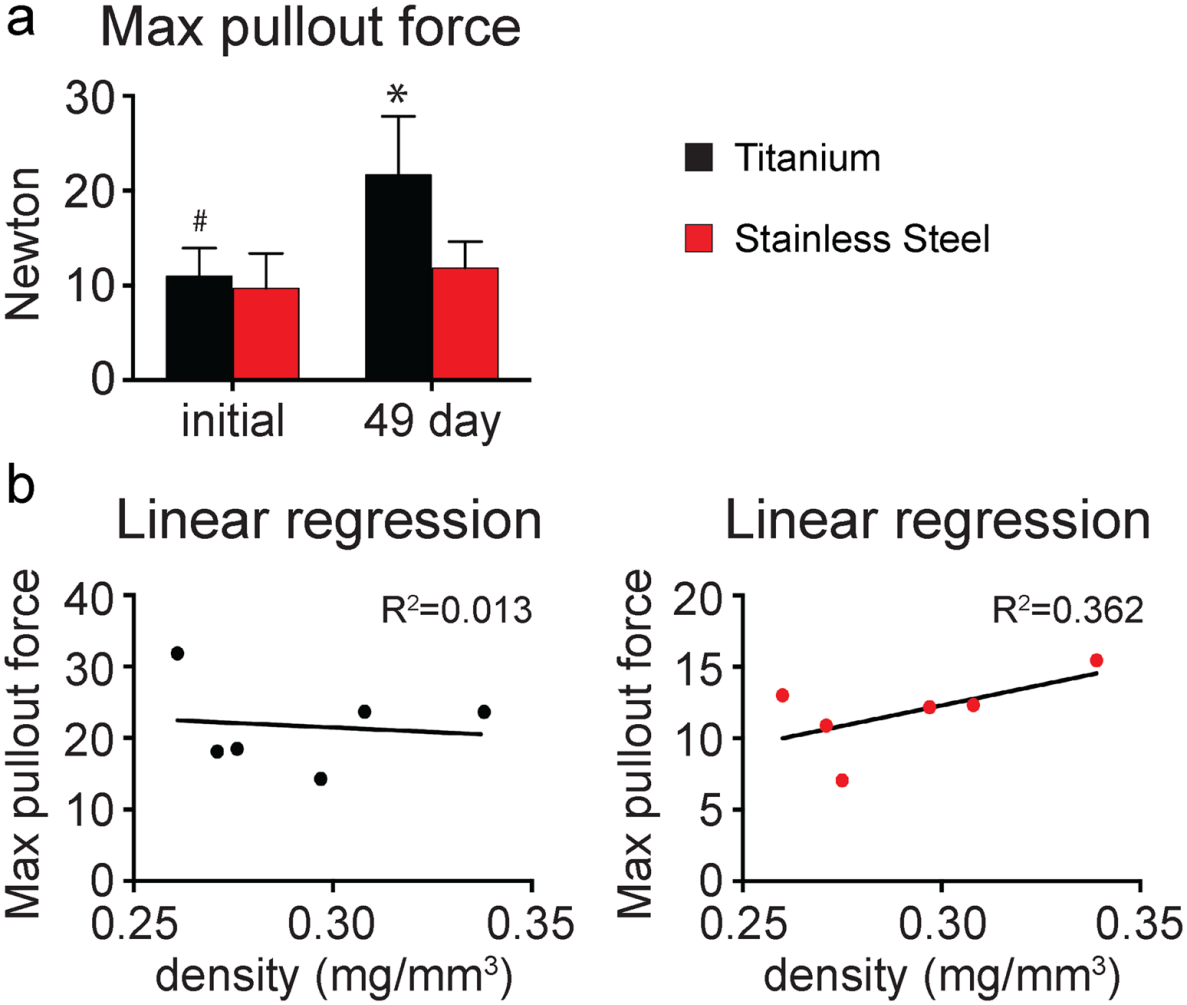
Integration strength. (**a**) Graph showing the max pullout force required to extract titanium and stainless steel implants before and after 7 weeks of culture in an osteogenic environment. Data represent averages ± SD (n = 6, paired and unpaired Student’s t-test; pound denotes significant difference between initial and 49 days; asterisk denotes significant difference between the titanium and stainless steel groups). (**b**) Linear regression analysis between the max pullout force measured and the densities of the decellularized bone scaffolds. Additional results are shown in Fig. S12 and Video S1.

Long-term stability of prosthetic implants is critical to achieving therapeutic efficacy. After implantation, a mechanical bond (primary stability) forms between the tissue and the implant, whose strength is determined by the implant design and the bone architecture at the site of implantation. As the tissue regenerates and remodels at the implantation site, a secondary, biological bond (secondary stability) forms between the tissue and the implant. Secondary stability is strongly influenced by the surface properties of the implant, and is critical for successful osseointegration^53,54^. In this study, pullout testing shows that primary stability is equal for Ti and SS implants, since the implant design and scaffold architecture are comparable (Fig. 5, Fig. S12, Video S1). In contrast, secondary stability is significantly higher in Ti implants (*p* value: 0.0224) (Fig. 5a), with the average maximum pullout force increasing by 97.91% for Ti implants and only 22.14% for SS implants. Linear regression analysis excludes any possible relationship between the max pullout force and the density of the scaffolds (Fig. 5b), confirming that the difference in secondary stability fully results from a disparate biological response to Ti and SS implants. The higher increase in pullout force observed for Ti implants correlates well with the greater tissue mineralization observed in response to this material (Fig. 4c). Previous studies have demonstrated a positive correlation between the presence of mineral deposits in the peri-implant region and the strength of the bone-implant interaction^55,56^, providing strong evidence that the higher mineral content in Ti samples directly correlates with the higher tissue-implant interaction strength. Collectively, these results are consistent with preclinical and clinical studies, showing that Ti implants form a direct anchorage to bone, while SS implants connect to the tissue via a continuous cell layer^17^.

**Figure 6:**
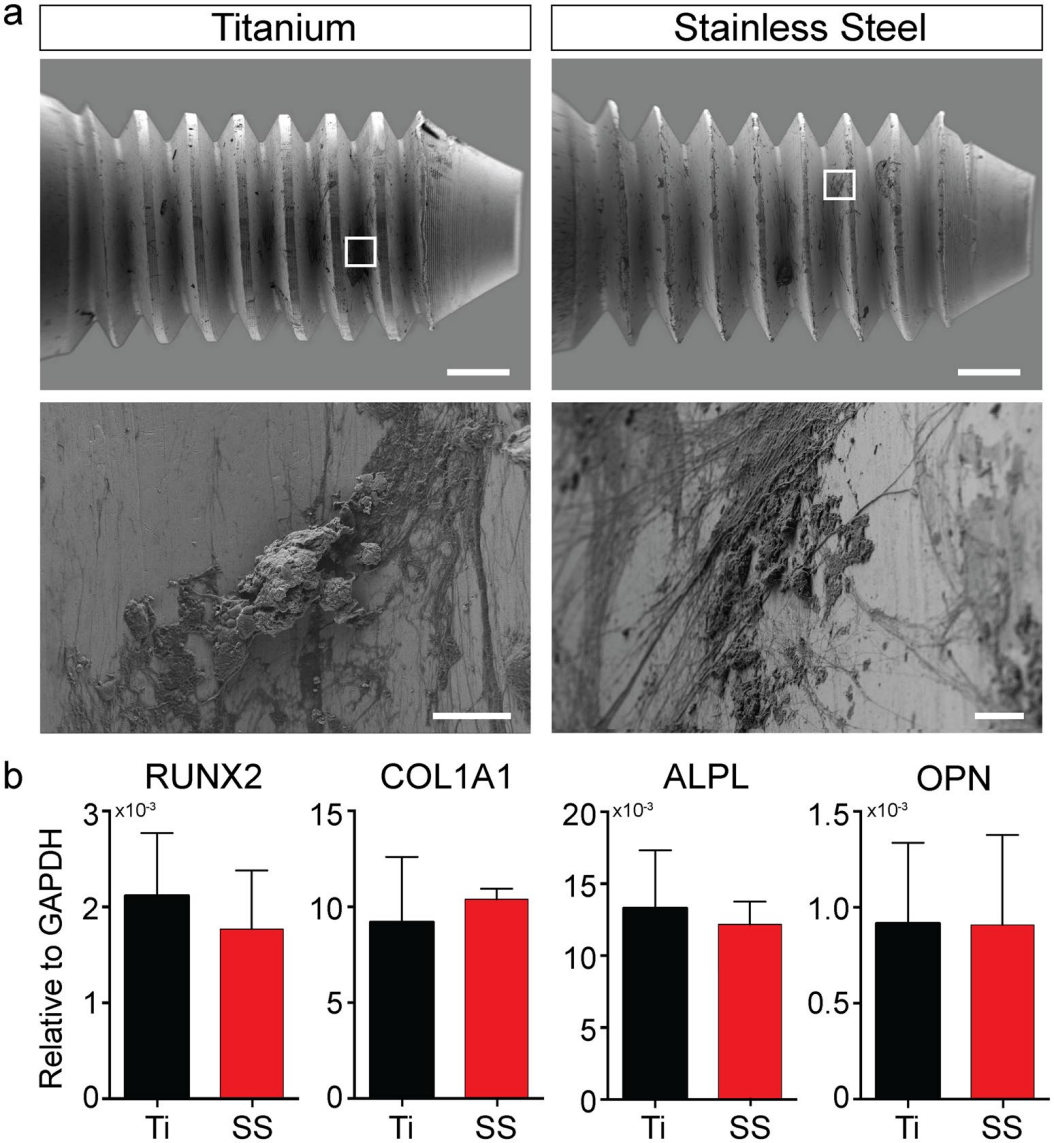
Post-pullout implant characterization. (**a**) Scanning electron micrographs of titanium and stainless steel implants after pullout showing residual biological material. Scale bar: 500 µm (top) and 10 µm (bottom). (**b**) Real-time PCR data showing the expression of runt-related transcription factor 2 (RUNX2), collagen type I alpha 1 (COL1A1), alkaline phosphatase liver/bone/kidney (ALPL), and osteopontin (OP) at the tissue-implant interface for titanium and stainless steel samples. (n = 3, unpaired Student’s t-test). Additional results are shown in Fig. S13.

An effective testing system should reproduce the interfacial phenomena responsible for tissue-implant integration and stability (i.e. extracellular matrix quantity and composition, and the deposition of calcium phosphate minerals), and allow for iterative design of implant systems that modulate tissue regeneration. In this study, after pullout testing, the implants were further characterized to analyze the surface characteristics and analyze the biological material remaining on the implants (Fig. 6, Fig. S13). SEM imaging show more residual cellular material on the surface of SS implants compared to Ti implants (Fig. 6a), additionally supporting the knowledge that SS implants connect to the bone tissue via a continuous cell layer^17^. Energy-dispersive X-ray spectroscopy (EDS) analysis confirms that calcium and phosphate are present on the surface of both Ti and SS implants after pullout (Fig. S13), implying that the testing platform can simulate the host physiological microenvironment, and can evaluate the material properties of new implant systems in vitro.

Studying the biological response of the cells in direct contact with an implant is critical for understanding the tissue-implant interaction processes that lead to osseointegration, but difficult to evaluate using 2D methods and animal models. In this study, we collected biological material from the implant surface after pullout testing to study the expression of prototypical osteogenic genes (Fig. 6b). The results show the expression of COL1A1 and OPN at the implant surface (Fig. 6b) differ from the sample bulk (Fig. 3e) in response to SS implants.

This could indicate a delay in osteogenic differentiation and tissue formation in response to SS implants. Alternatively, it may indicate a pathological response to an aberrant surface^57,58^, in which higher expression of the extracellular matrix mineralization initiators COL1A1 and OPN produces negligible increase in mineral density and no improvement in secondary fixation strength after culture. OPN is also involved in other biological processes, including chemotactic cell recruitment, mechano-transduction, adipogenic differentiation, bone resorption, and regulation of glycolytic metabolism^59,60^. It is plausible that SS implants stimulate a chronic COL1A1 and OPN expression, which result in the formation of an atypical extracellular matrix that does not mineralize as readily.

The limitation of this study is that it includes only a single cell type and recapitulates only localized phenomena of the osseointegration process that occurs in vivo. While the study reproduces unique changes in extracellular matrix production, mineralization, and macroscale phenotypic changes (e.g., thickening of trabecula during culture), it does not include cells involved in inflammation, neovascularization, and bone resorption, which are recognized to play an important role during osseointegration in vivo. Also, this study does not consider the microbioma that can affect negatively the regeneration process and lead to implant failure. Finally, this study does not take into account the systemic hormonal status of the patient, considering for example that low estrogen levels are associated with poor osseointegration in osteoporotic bone^61^, as well as other macroscale effects such as the role of mechanical loading, synovial fluid, blood supply, and surrounding soft tissues, as well as the anatomical location in which an implant is placed. In light of these limitations, future work will include inflammatory and angiogenic cells that are known to play an important role in the osseointegration process^62^, take into consideration the systemic hormonal status, and the role of the microbiome, which can lead to inflammatory destruction of peri-implant tissue^63^, and test the effect of drugs that can promote bone regeneration around an implant. Also, since loading can affect bone regeneration at the peri-implant region^64^, we will use compressive bioreactors providing physical stimulation to the bone-implant system and study the relation between loading, new tissue formation, and implant stability. Despite these limitations, our bone-implant platform can be used to study the role of genetic polymorphisms and age on the osseointegration process by using cells derived from individuals with different race, gender, age, and other pre-existing health conditions.

In summary, our platform reproduces fundamental elements of the osseointegration process, across a range of molecular, cellular, and tissue scale phenomena, including the interfacial phenomena that underlie mineralization and formation of a biological bond between the tissue and the implants in vivo. Importantly, the platform recapitulates the stronger osseointegration properties observed for Ti implants in vivo^17^, supporting its application to evaluate the integration potential of new implants and biomaterials to create fundamental knowledge and predict clinical outcomes.

## Conclusions

We have devised a novel, biomimetic, human bone platform that facilitates testing of implant systems within a context that closely resembles the native tissue environment. Our platform provides a significant benefit over available methods for testing new implants and for creating a fundamental understanding of the osseointegration process, since it allows identification of the molecular mechanisms and cellular events associated with integration, or failure, of prosthetic implants in humans. This study is a proof of concept, which demonstrates that 3D stem cell models of human bone can reproduce complex elements of the tissue-implant interaction process in vitro. We have found that molecular pathways involved in cell metabolism, growth, communication and osteogenic differentiation are differentially regulated in response to Ti and SS implants, likely contributing to the relative osseointegration properties of these two materials. The system can be used to model different phases of the osseointegration process, using mono- or co-culture systems, such as the inflammatory response and the bone remodeling process taking place at the peri-implant region, as well as the cellular crosstalk that occurs during the healing process. Furthermore, our platform creates the possibility of testing the antibacterial properties of new implant surfaces and the therapeutic potential of drug-delivering implant systems. In addition to enabling a better understanding of the tissue response to implants, this platform will allow researchers to study the behavior of new materials and surface modifications in a simulated patient-specific biological environment. The technology shows strong potential to advance development of superior orthopedic implants and biomaterials, and to reduce the need for animal testing.

## Materials and methods

### Implants

Titanium and stainless steel M2 implants, 2 mm in diameter and 7 mm in length, with a self-tapping edge 1.5 mm long, a thread pitch of 0.4 mm, an outer thread diameter of 2 mm, and thread depth of 0.225 mm, were fabricated by Engemikroteknik AB (Vittsjö, Sweden). The implants were manufactured from medical-grade materials (ASTM F67 GR4 titanium and 316L stainless steel) using an ERGSTE 1.441 LA lathe (Citizen S12).

### Optical profilometry

The surface roughness of the Ti and SS implants was measured on the apical regions of the implants using a Wyko NT-1100 (Veeco Instruments, Inc.) optical profiler in vertical scanning interferometry (VSI) mode. The measurements were done on three different sites along the implant heads, parallel to the axis of the screw, and expressed in terms of the arithmetical mean deviation of the profile (Ra).

### Scanning electron microscopy and energy-dispersive x-ray spectroscopy

Scanning electron microscopy (SEM) was used to characterize the Ti and SS implant surfaces before insertion, and following pullout testing after 7 weeks of culture in an osteogenic environment. The samples were placed onto SEM stubs using carbon tape, and images were acquired on a Zeiss Merlin (Zeiss) equipped with a high efficiency secondary electron detector and operated at an acceleration voltage of 3 kV and a probe current of 100 pA.

Following SEM imaging, elemental mapping (energy dispersive X-ray spectroscopy, EDS) was carried out on an X-Max 80 mm^2^ silicon drift EDS detector, at 20 keV, and a probe current of 700 pA. The data were analyzed with Aztec analysis software (AZTEC 3.3 SP1, Oxford Instruments).

### Scaffolds

Biomaterial scaffolds were prepared as previously described^15^. Briefly, plugs of trabecular bone (8 mm diameter) were drilled from the proximal and distal regions of metacarpal and metatarsal calf bones. Bone plugs were washed under a high-pressure water stream to remove the blood and bone marrow, and decellularized by serial incubation in (1) Dulbecco's phosphate-buffered saline (DPBS) containing 0.1% (w/v) trypsin-ethylenediaminetetraacetic acid (EDTA) at room temperature (RT) for 1 h, (2) 10 mM Tris containing 0.1% EDTA (w/v) at 4 °C for 12 h, (3) 10 mM Tris containing 0.5% (v/v) sodium dodecyl sulfate (SDS) at RT for 24 h, and (4) 10 mM Tris containing 100 U/mL of DNase and RNase (both from Sigma) at 37 °C for 6 h. Following decellularization, the bone plugs were washed in DPBS, dehydrated in research-grade pure ethanol, freeze-dried overnight, and cut to 5 mm in length. Finally, the scaffolds were weighed and measured to calculate sample density using the following formula (1):

1 $$ {\text{density}} = {\text{weight}}/\uppi \times \left( {{\text{scaffold}}\,{\text{diameter}}/2} \right)^{2} \times {\text{scaffold}}\,{\text{height}}. $$

Scaffolds in the range of 0.25–0.45 mg/mm^3^ were finally selected and matched for each analysis (Table S1).

### Cells

Human iMSCs (line 1013A) were derived from a healthy male donor and characterized as previously described^15^. At passage 5, cells were cultured in expansion medium consisting of high-glucose KnockOut Dulbecco’s Modified Eagle’s Medium (KO-DMEM; Gibco), 10% (v/v) HyClone fetal bovine serum (FBS; GE Life Sciences), beta-fibroblast growth factor (1 ng/ml; R&D systems, Minneapolis, MN), GlutaMax (1X; Gibco), nonessential amino acids (1X; Gibco), 0.1 mM β-mercaptoethanol (Gibco), and antibiotic–antimycotic (1X; Gibco). At confluence, cells were detached using trypsin/EDTA (0.25%; Gibco), counted with a hemocytometer, and suspended in expansion medium at a density of 20 × 10^6^ cells/ml prior seeding.

### Engineering the bone-implant platform

Decellularized bone scaffolds were placed in a customized silicone rubber holder to create a perpendicular thread in the center of the scaffolds, using a tap holding stand and a M1.6 tap (1.6 mm in diameter), and cleaned using a compressed gas duster. Ti and SS implants were sterilized in a Tuttnauer Benchtop Autoclave 2540EP (Tuttnauer), at 121ºC for 20 min, and then anchored manually into the scaffolds. After implantation, the scaffold-implant constructs were sterilized in 70% (v/v) ethanol at RT overnight and conditioned in expansion medium at 37ºC for 24 h. Prior to seeding, the scaffold-implant constructs were blot-dried using autoclaved Kimwipes (Fisher Scientific), and placed upside down (i.e. with the implant head sitting on the floor as shown in Fig. S3) in ultra-low attachment plates (Fisher scientific) to maximize cell attachment. Finally, 100 µl aliquots of the cell suspension containing 2 × 10^6^ cells were added to each scaffold-implant construct using an optimized droplet seeding technique. Following seeding, the samples were placed in a humidified environment at 37 °C for 3 h to let the cells attach to the scaffolds, and then cultured in expansion medium overnight. The following day, the expansion medium was collected, and unattached cells counted using a hemocytometer to estimate the seeding efficiency using the following Eq. (2):

2 $$ {\text{Seeding}}\,{\text{efficiency}} = \frac{{{\text{Number}}\,{\text{of}}\,{\text{seeded}}\,{\text{cells}} - {\text{Number}}\,{\text{of}}\,{\text{unattached}}\,{\text{cells}}}}{{{\text{Number}}\,{\text{of}}\,{\text{seeded}}\,{\text{cells}}}} \times 100\% $$

The samples were finally cultured in a osteogenic environment consisting of high-glucose DMEM (Thermo Fisher Scientific) supplemented with 10% (v/v) HyClone FBS (GE Life Sciences) and the osteogenic factors L-ascorbic acid (50 µM; Sigma), dexamethasone (1 µM; Sigma), and β-glycerophosphate disodium salt (10 mM; Sigma-Aldrich). Samples were culture under these conditions for 7 weeks to ensure the formation of a thick, mature tissue around the implants. Medium was changed 3 times per week throughout the experiment and medium aliquots stored for content analysis. At the end of the culture, the samples were washed in DPBS and processed to study the tissue response to the implants using a combination of biochemical assays, imaging and histological methods, high-resolution characterization techniques, and biomechanical testing.

### Cell distribution, viability and growth

Cell distribution, viability and growth were monitored using the LIVE/DEAD assay (Molecular Probes) following the manufacturer’s instructions. Briefly, 1 day and 7 weeks after culture in osteogenic conditions, samples were harvested, washed in DPBS, and incubated at 37 °C in the dark with a solution of calcein AM (2 mM) and ethidium bromide (4 mM) in DPBS for 1 h. Following incubation, the samples were washed with DPBS, and then placed in RPMI (medium without red phenol; Lonza) for imaging. Fluorescence images were taken with an Olympus IX71 (Olympus) and assembled together into mosaics using ImageJ (National Institute of Health) equipped with the MosaicJ and TurboReg plugins. Confocal images were taken with the Axiovert 200 M microscope (Carl Zeiss AG) mounted with LSM 5 Pa exciter using the LSM 5 Pa software under defined settings.

### Histology of non-demineralized samples

After 7 weeks of culture in osteogenic conditions, samples were histologically examined to study tissue formation and composition, and the characteristics of tissue-implant interface. Briefly, samples were fixed in 4% (v/v) paraformaldehyde (PFA) in PBS at 4 °C for 24 h, dehydrated with ethanol solutions, and immersed into methyl methacrylate as previously described^65^. Then, embedded samples were cut longitudinally into 400 µm sections using an IsoMet™ low-speed diamond blade (Buehler), polished using 1200-grit paper (Buehler, MicroCut™ disc), glued to plastic slides (Exakt, plastic slides 25 × 75 × 1 mm), ground with the Exakt grinder (Exakt) equipped with a 600-grit abrasive paper (Exakt, WS Flex 18 C), polished on an EcoMet™ 30 twin manual grinder (Buehler) in two steps using a 800-grit and a 1200-grit silicon carbide abrasive paper (Buehler, MicroCut™), fine-polished using a clear coat scratch remover (Kit, Scratch out®), and stained with Stevenel’s blue and van Giesson picrofuchsin solution. Images were taken with an Olympus IX71 and combined in mosaics using MosaicJ (National Institutes of Health).

### Histology of demineralized samples

The presence of collagen fibers in the matrix and deposition of bone glycoproteins were studied on demineralized samples following pullout testing. Samples were washed in DPBS, fixed in 4% (v/v) PFA in PBS at 4 °C for 24 h, and then decalcified in Immunocal (Decal Chemical Corp.) for 48 h at RT as previously reported^66^. After decalcification, the samples were dehydrated through graded concentrations of ethanol prior to paraffin embedding. Then, 5 µm tich Sects. (5 µm in thickness) were then cut, mounted on glass slides, and stained with Hematoxylin and Eosin and Masson's Trichrome. To study deposition of bone matrix proteins, sections were deparaffinized at 60 °C for 30 min followed by incubation in CitriSolv (twice for 5 min), rehydrated with ethanol, incubated in deionized H_2_O (3 times for 2 min), and washed in DPBS for 5 min. The sections were then incubated in citrate buffer (pH 6) at 90 °C for 30 min for antigen retrieval, washed in deionized H_2_O for 5 min, and incubated with 3% H_2_O_2_ in methanol for 30 min to block the endogenous peroxidase activity. Following a wash in DPBS for 5 min, sections were incubated with 1% normal horse serum in DPBS (Vectastain ABC kit Elite) to block the non-specific binding and stained overnight at 4 °C in a humidified chamber with primary antibodies (all purchased from Millipore and diluted 1:500 in DPBS) against osteopontin (rabbit polyclonal anti-osteopontin, #AB1870), osteocalcin (rabbit polyclonal anti-osteocalcin, #AB10911) and bone sialoprotein (rabbit polyclonal anti-BSP II, #AB1854). Specific antigen detection was performed using the biotinylated secondary antibody and biotin/avidin complex (Vectastain ABC kit Elite) diluted in DPBS via incubation with 3,3′-diaminobenzidine peroxidase substrate for 5 min (Vector DAB kit). Sections were then counterstained with hematoxylin (Hematoxylin 7211, Richard-Allan Scientific), dehydrated with a graded series of ethanol washes (50%, 70%, 95%, twice 100%; each for 2 min), incubated with Citrisolv (twice for 5 min), dipped into xylene, and sealed with coverslips (Fisherbrand) using the Permount mounting media (Fisher Chemicals Scientific). Negative controls were performed following the same procedure but omitting either the primary or secondary antibody incubation. Micrographs of slides were taken with an Olympus IX71 and combined in mosaics using MosaicJ (National Institutes of Health).

### RNAseq analysis

Following implant pullout, RNA was extracted from the samples to study the molecular response to the implants via differential gene expression analysis. Briefly, samples were lysed in Trizol buffer (Qiagen) containing 20% (v/v) chloroform, and total RNA was isolated using the RNeasy Mini Kit (Qiagen) according to manufacturer’s instructions. Following isolation, RNA samples were quantified using a fluorescent-based assay to accurately determine whether sufficient material was available for library preparation and sequencing. RNA sample size distributions were profiled using the Agilent 2100 BioAnalyzer (Agilent Technologies) to assess sample quality and integrity. RNA sequencing libraries were prepared using the TruSeq Stranded mRNA Library Preparation Kit in accordance with the manufacturer’s instructions. Briefly, 500 ng of total RNA were used for purification and fragmentation of mRNA. Purified mRNA was converted to double strand cDNA, which was then adenylated, ligated to Illumina sequencing adapters, and amplified by 10 cycles of polymerase chain reaction. Final libraries were evaluated using the PicoGreen assay (Life Technologies) and the Agilent 2100 BioAnalyzer (Agilent Technologies), and were sequenced on an Illumina HiSeq2500 sequencer using 2 × 125 bp cycles. Reads were aligned to the GRCh37 human reference (chr1-22, X, M, and Y) using STAR 2.5.2a. Quantification of genes annotated in Gencode v18 was performed using the featureCounts (v1.4.3-p1). Quality control analysis was performed with Picard (v1.83) and RSeQC (v2.6.1). Normalization of gene counts and differential expression were performed with DESeq2 (v1.18.1).

Enrichment of DEGs was carried out in R version 3.6.0 using the package Goseq (version 1.36.0)^67^ against the Gene Ontology (GO-db_3.8.2) database^23^. Ensembl gene IDs on hg19 were used as gene identifiers. Goseq corrects for gene length bias followed by the 'Wallenius' method to compute the enrichment scores and p-values. The raw p-values were corrected for multiple testing using Benjamini–Hochberg correction resulting in adjusted-p-values. Enrichment analyses was also carried out using the same dataset against the KEGG (KEGGREST_1.24.0)^25^ and Reactome (reactome.db_1.68.0) pathway database^26^. The list of GO terms was reduced based on semantic similarity using the REVIGO Web server^24^.

Functional analysis of RNAseq data to search for enriched signaling pathways was conducted through the use of IPA (QIAGEN Inc., https://www.qiagenbioinformatics.com)^27^.

Functional classification of differentially expressed genes involved in osteoblast differentiation and bone formation was conducted using the Gene Ontology Resource filtering for the GO term ‘*ossification*’ and narrowing the search to include only the UniProtKB as a data source. To investigate possible interaction among proteins encoded by differentially expressed human genes involved in the ossification process, the search tool STRING^38^ was used to mine for statistically relevant co-occurrences of genes.

### Real-time polymerase chain reaction

The expression of mesodermal genes in response to Ti and SS implants was studied via real-time PCR as previously^15^. After pullout, whole tissue samples and extracted implants were lysed in Trizol buffer (Qiagen) and total RNA was isolated using the RNeasy Mini Kit (Qiagen). After isolation, total RNA was reverse-transcribed with random hexamers using the GoScript™ Reverse Transcription System (Promega, Madison, WI). The expression of the collagen, type I, alpha 1 (COL1A1; Hs00164004_m1), osteopontin (OPN; Hs00959010_m1), runt-related transcription factor 2 (RUNX2; Hs00231692_m1), liver/bone/kidney alkaline phosphatase (ALPL; Hs01029144_m1), integrin-binding sialoprotein (IBSP; Hs00173720_m1), sex-determining region Y-box 9 (SOX9; Hs00165814_m1), peroxisome proliferator-activated receptor gamma (PPARG; Hs01115513_m1), and the housekeeping gene glyceraldehyde 3-phosphate dehydrogenase (GAPDH; Hs02758991_g1) was determined using the StepOnePlus PCR System cycler (Applied Biosystems) using the TaqMan Universal PCR Master Mix and TaqMan Gene Expression Assays (Applied Biosystems). Expression of target genes was normalized to the expression level of GAPDH.

### Bioanalyte analysis

Cell metabolic activity was monitored weekly by measuring the content of glucose and lactate in the medium, and the medium pH. Briefly, frozen aliquots of culture medium were thawed on ice and 65 µl used to conduct the measurements using the Vi-CELL MetaFLEX™ analyzer (Beckman Coulter). Fresh osteogenic medium was used as control for all analyses.

### Lactate dehydrogenase

Cell death was monitored weekly by measuring the content of lactate dehydrogenase (LDH) in the culture medium using the Pierce™ LDH Cytotoxicity Assay Kit (Thermo Scientific) according to the manufacturer’s protocol. Briefly, 50 µl aliquots of cell culture medium was incubated at RT with 50 µl aliquots of reaction mixture containing lactate, diaphorase and tetrazolium salt (INT) for 30 min. The reaction was stopped with 50 µl of Stop Solution and the absorbance of red formazan product was read at 490 nm using the plate reader SYNERGYMx (BioTek) equipped with Gen 5 1.09 software. The level of formazan formation is directly proportional to the amount of LDH released in the medium. The activity of lactate dehydrogenase was calculated by subtracting background readings (absorbance read at 680 nm) and expressed in absorbance values “A490-680 nm”.

### Multiplex immunoassay

The release of trophic factors was investigated using the ProcartaPlex™ Multiplex Assay (Invitrogen) as previously described^68^. On day 1, week 3 and week 7 of culture in an osteogenic environment, aliquots of media were collected, vortexed and centrifuged at 10,000×*g* for 10 min to remove particulates. Thus, aliquots of cell culture supernatants, controls and standards were added to a custom ProcartaPlex™ plate to assay for vascular endothelial growth factor A (VEGF-A), interleukin 2 (IL-2), interleukin 6 (IL-6), interleukin 8 (IL-8), monocyte chemoattractant protein 1 (MCP-1), and macrophage inflammatory protein 1 beta (MIP-1β). Following detection, the analyte contents were measured using the MAGPIX™ (Invitrogen) platform equipped with the Luminex™ Acquisition Software (Thermo Fisher Scientific). Fresh osteogenic media was used for background subtraction. Data are expressed as fg/ml.

### Alkaline phosphatase activity

The release of alkaline phosphatase (ALP) into the culture medium was studied weekly using the Alkaline Phosphatase Colorimetric Assay Kit (BioVision) as previously described^16^. Briefly, 80 µl of culture medium were added to a transparent, flat-bottom, 96-well plate, mixed with 50 µl of 5 mM paranitrophenylphosphate (*p*NPP) diluted in ALP Assay Buffer, and incubated at RT for 2 h. The reaction was stopped with 20 µl of Stop Solution and absorbance was read at 405 nm using the plate reader SYNERGYMx (BioTek) equipped with Gen 5 1.09 software. The zctivity of ALP was calculated by subtracting background readings and using the *p*NPP standard curve. Data are expressed as nM/h.

### Osteocalcin

The release of osteocalcin was studied weekly using the Gla-type Osteocalcin EIA kit (Takara) as previously described^16^. Briefly, the Gla-type Osteocalcin EIA plate was incubated at RT with 100 µl aliquots of culture medium for 2 h, 100 μl of Horseradish Peroxidase Conjugated Osteocalcin Antibody Solution for 1 h, and 100 μl of Substrate Solution for 15 min. The reaction was stopped with 100 µl of Stop Solution and absorbance was read at 450 nm using the plate reader SYNERGYMx (BioTek) equipped with Gen 5 1.09 software. The amount of released osteocalcin was estimated by subtracting background readings and using the osteocalcin kit standard curve. Data are expressed as fg/ml.

### Microcomputed tomography

Microcomputed tomography was used to study tissue mineralization and determine the percentage of bone-implant contact area. Before cell seeding and 7 weeks after culture in an osteogenic environment, samples were washed in PBS, fixed in 4% (v/v) PFA in PBS at 4 °C for 24 h, and then scanned in the high-resolution SkyScan microCT system (SkyScan 1172; Kontich, Belgium) using a 10-MP digital detector at 10 W of energy (80 kV and 124 mA), a pixel size of 6 microns, a rotation step of 0.4 degrees with ×8 frame averaging, and a scan rotation of 180 degrees. After scanning, the radiographs were reconstructed using the NRecon v1.7.3.0 software (Bruker micro-CT) using GPU acceleration. Gaussian smoothing was applied with a 2 voxel radius, and ring artifact reduction set to 7 pixels. Beam hardening correction was set to 40%. Bone volume fraction (BV/TV), trabecular number (Tb.N), and trabecular thickness (Tb.Th) were determined with the structural reconstruction.

### Time-of-flight secondary ion mass spectrometry

Mineral deposition at the tissue-implant interface was investigated via Tof–SIMS. Briefly, sections of resin-embedded samples (produced as described above in the Histology of non-demineralized samples section) 7 weeks after culture in an osteogenic environment were imaged using the nanoTOF II (Physical Electronics) with 20 kV Ga^+^ as primary analysis ions under ultra-high vacuum (~ 10^−6^ Pa). Prior to analysis, the sample surface was sputtered with a 20 kV Ar^+^ Giant Cluster Ion for 1 to 5 min to remove any possible surface contamination by removing a superficial layer of about 10 to 50 nm. During SIMS measurement, the surface charge was neutralized with low energy electron gun and gas gun (Ar^+^). Spectra and ion images were recorded over analysis areas of 600 × 600 µm^2^ and 200 × 200 µm^2^. The data were analyzed using the TOF-DR software (Physical Electronics). Samples without cells were used as controls.

### Pullout test

The bone-implant interaction strength was measured via pullout test. Briefly, prior to cell seeding and after 7 weeks of culture in osteogenic medium, samples were clamped using customized fixtures to exert an axial tensile load without torque application. To prevent samples from slipping, sand paper (Wetordry TRI-M-ITE 220 413Q) was applied to the grip edges of the clamping unit. Pullout tests were performed at a rate of 60 mm per minute and with a total displacement distance of 10 mm using the Instron DynaMite 8841 tester (Instron) with a 1000 N capacity. Load and displacement values were recorded in 0.1-mm increments, and the maximum load generated during pullout was defined as the maximum pullout strength and expressed in “Newtons”.

### Image processing and generation

Image size, level and background were adjusted in Adobe Photoshop CC (Adobe Systems Incorporated) to improve viewing. Images were combined into figure panels using Adobe Illustrator CC (Adobe Systems Incorporated).

### Statistical analysis

Statistical analysis was conducted in GraphPad Prism 6 version 6.0e (GraphPad Software, Inc.). Unpaired Student’s t-test with Bonferroni post-hoc test was used to compare the Ti and SS groups. Paired Student’s t-test was used to compare the same samples before and after treatment. Results are shown as means ± standard deviations. Differences between the mean values for each comparison were considered statistically significant when the *p* value was < 0.05.

### Ethics statement

All methods were carried out in accordance with relevant guidelines and regulations. All experimental protocols were approved by The New York Stem Cell Foundation Research Institute Committee. Consent to use the cells for research was obtained by Columbia University as part of an IRB-approved protocol. Cells were shared with NYSCF as deidentified. Therefore, no additional consent by NYSCF was required.

## Supplementary information

Supplementary information.

Supplementary information.

Supplementary information.

Supplementary Table S2.

Supplementary Table S3.

Supplementary Table S4.

Supplementary Video S1.

## Data Availability

The datasets generated during and/or analyzed during the current study are available from the corresponding author (G.M.d.P).
