# Supplementary material for: A biomimetic engineered bone platform for advanced testing of prosthetic implants
Source: Sci Rep. 2020 Dec 17;10:22154. doi: 10.1038/s41598-020-78416-w (PMC7747643; doi:10.1038/s41598-020-78416-w)
Supplement: Supplementary file 1 — Supplementary information. [file 41598_2020_78416_MOESM1_ESM.pdf]

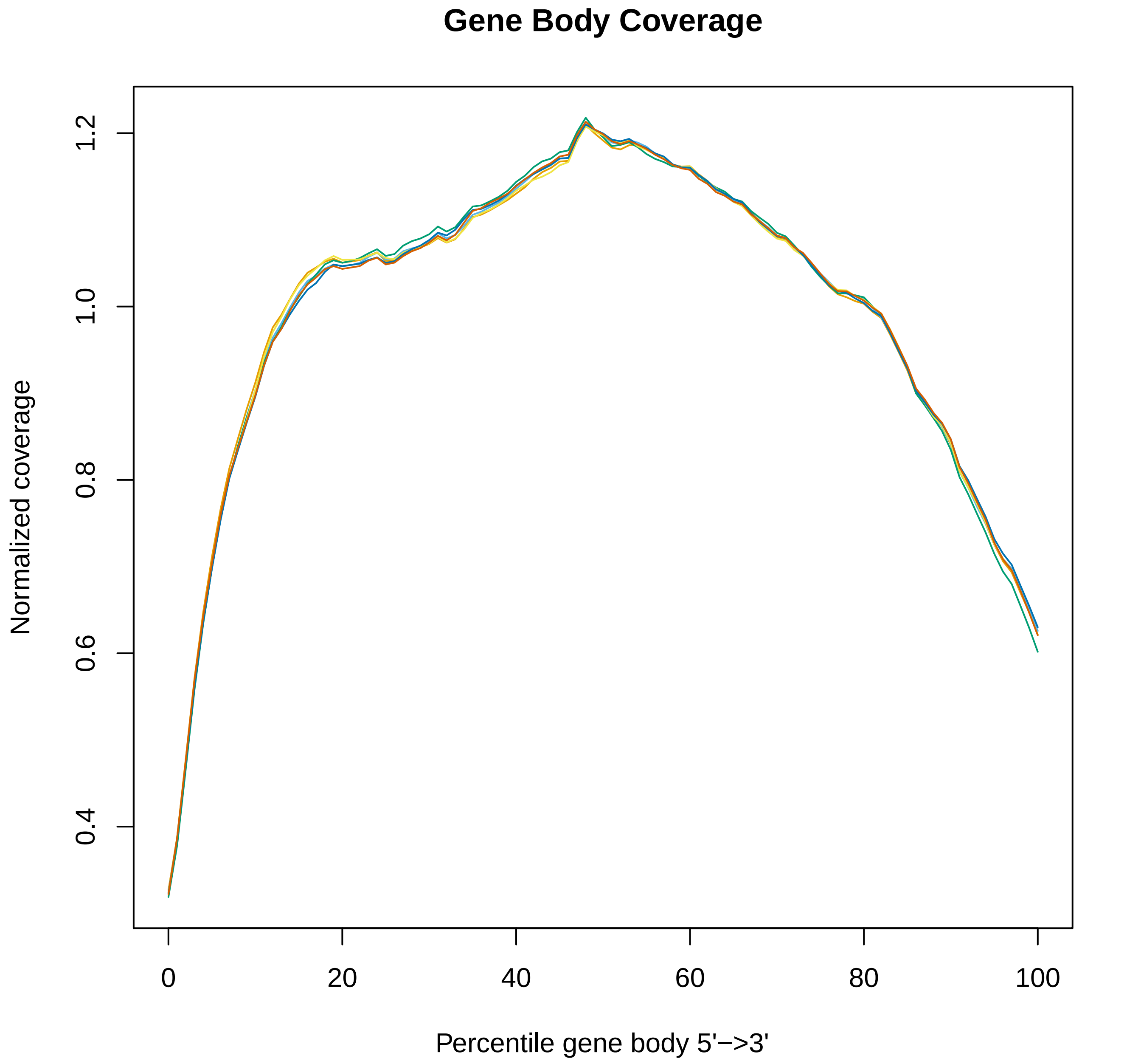
Gene Body Coverage
Normalized coverage
0.4
0.6
0.8
1.0
1.2
0
20
40
60
80
100
Percentile gene body 5'−>3'

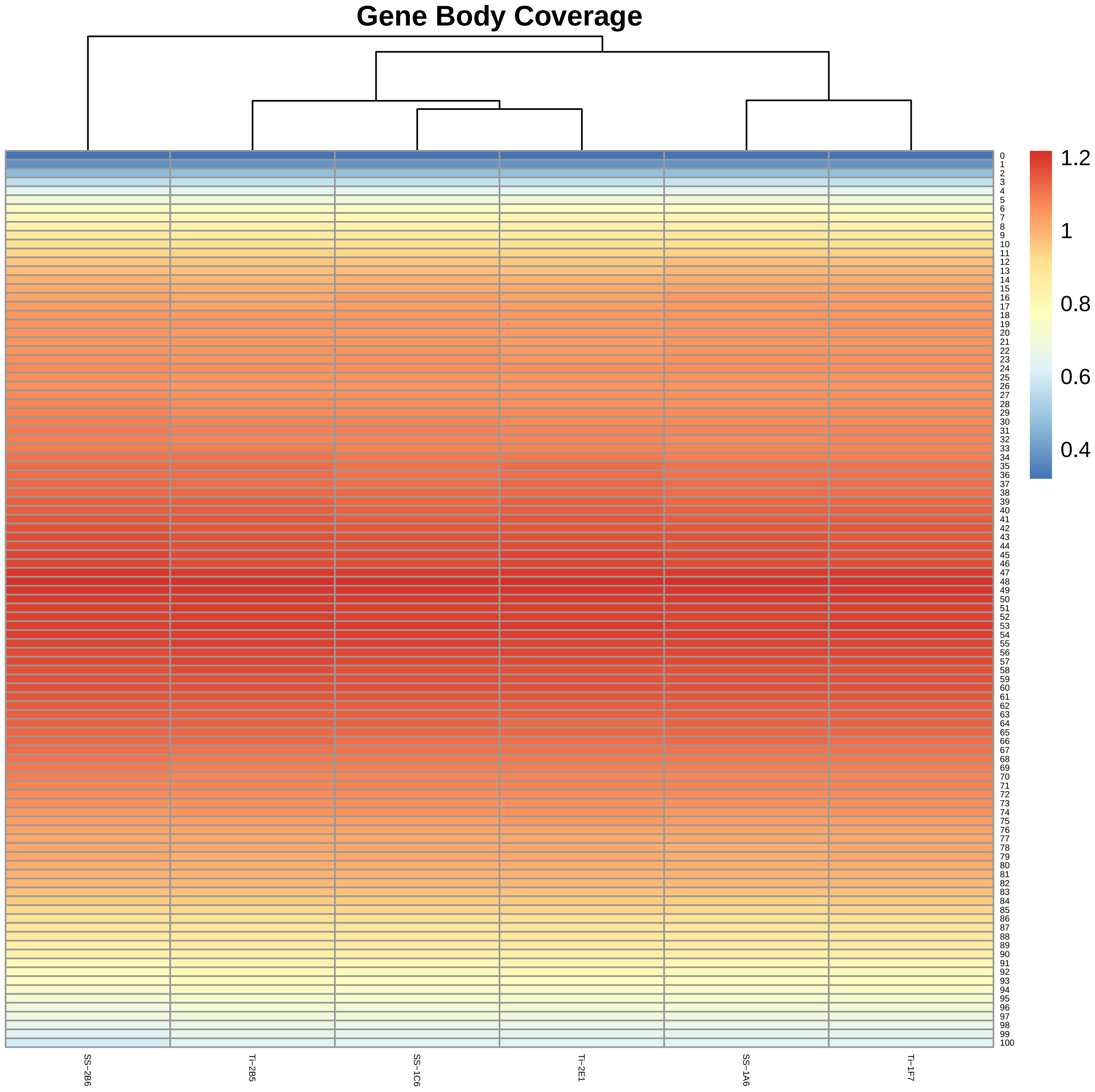
Gene Body Coverage
0 1 2 3 4 5 6 7 8 9 10 11 12 13 14 15 16 17 18 19 20 21 22 23 24 25 26 27 28 29 30 31 32 33 34 35 36 37 38 39 40 41 42 43 44 45 46 47 48 49 50 51 52 53 54 55 56 57 58 59 60 61 62 63 64 65 66 67 68 69 70 71 72 73 74 75 76 77 78 79 80 81 82 83 84 85 86 87 88 89 90 91 92 93 94 95 96 97 98 99 100
1.2
1
0.8
0.6
0.4
SS−2B6
Tr−2B5
SS−1C6
Tr−2E1
SS−1A6
Tr−1F7

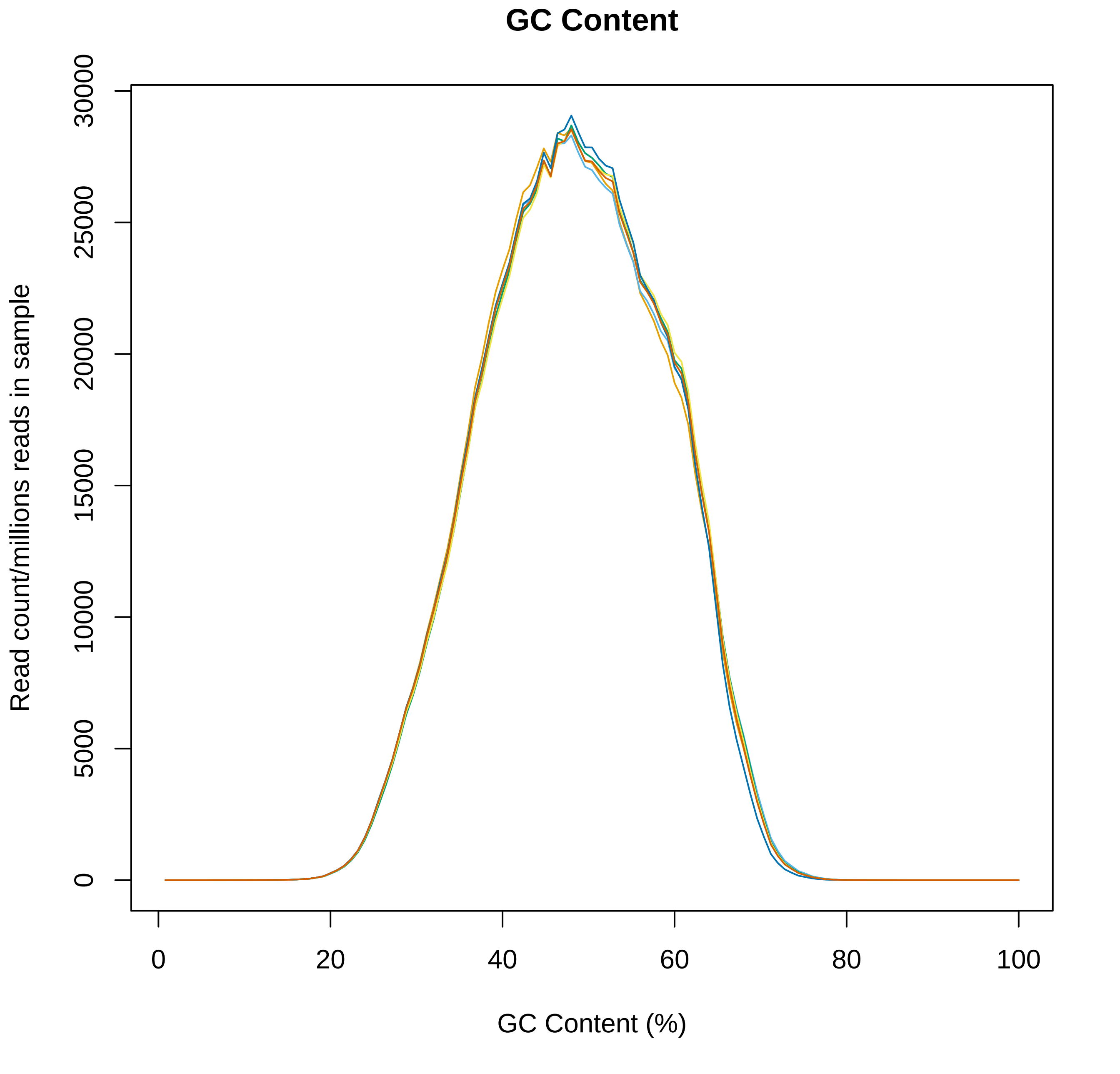

GC Content
Read count/millions reads in sample
0
5000
10000
15000
20000
25000
30000
0
20
40
60
80
100
GC Content (%)

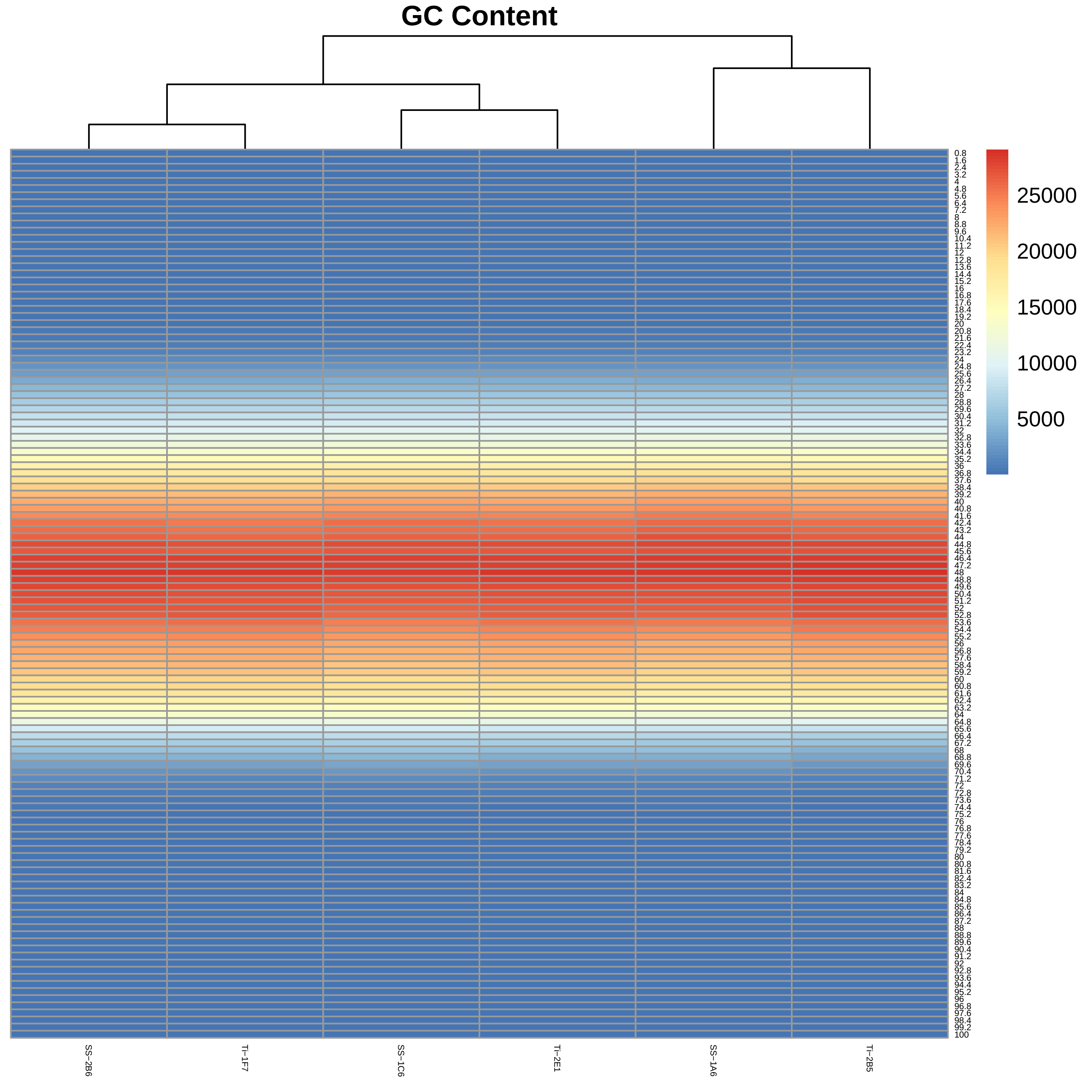

GC Content
25000
20000
15000
10000
5000
SS−2B6
Ti−1F7
SS−1C6
Ti−2E1
SS−1A6
Ti−2B5

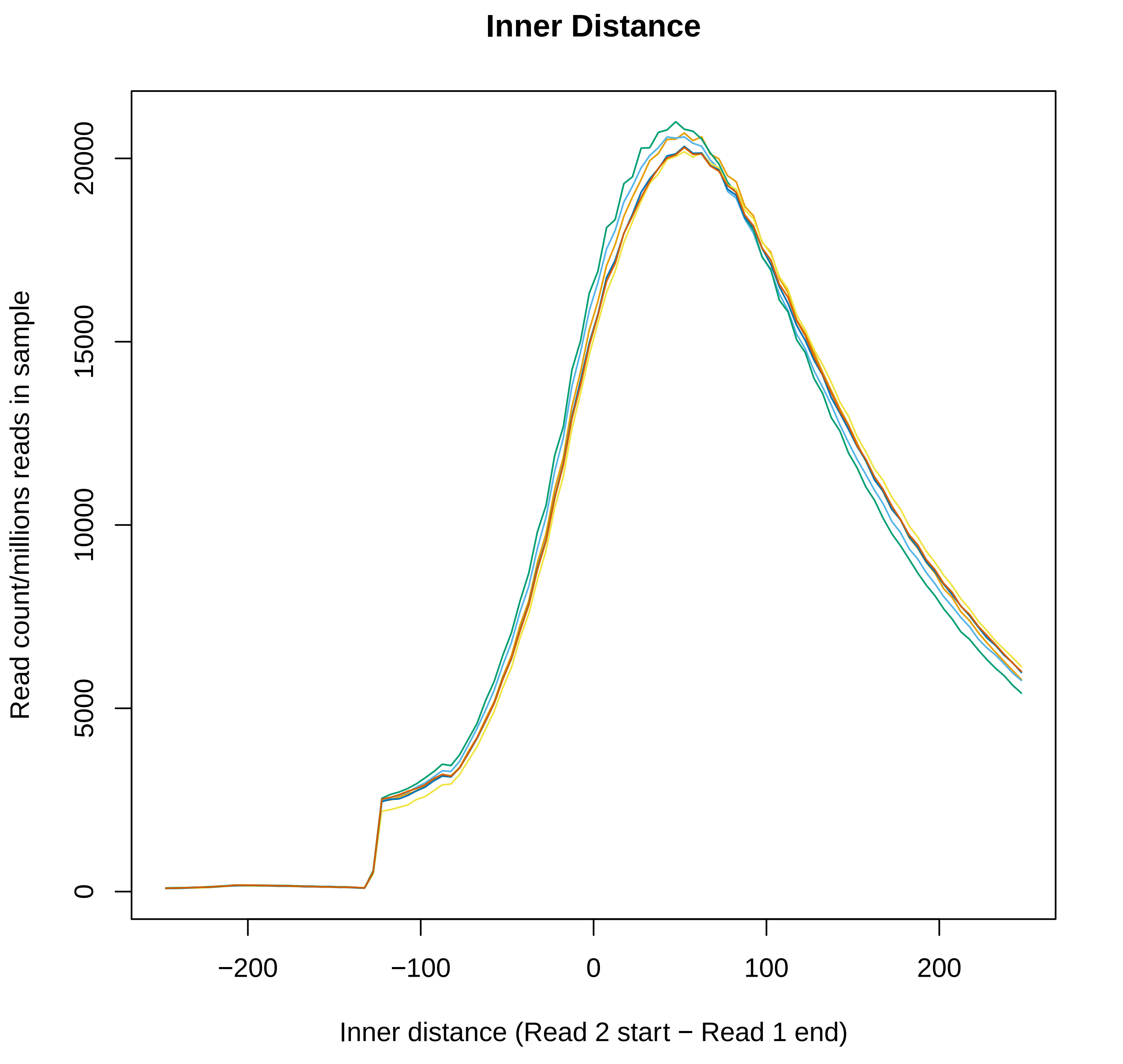

Inner Distance
Read count/millions reads in sample
0
5000
10000
15000
20000
−200
−100
0
100
200
Inner distance (Read 2 start − Read 1 end)

Inner Distance

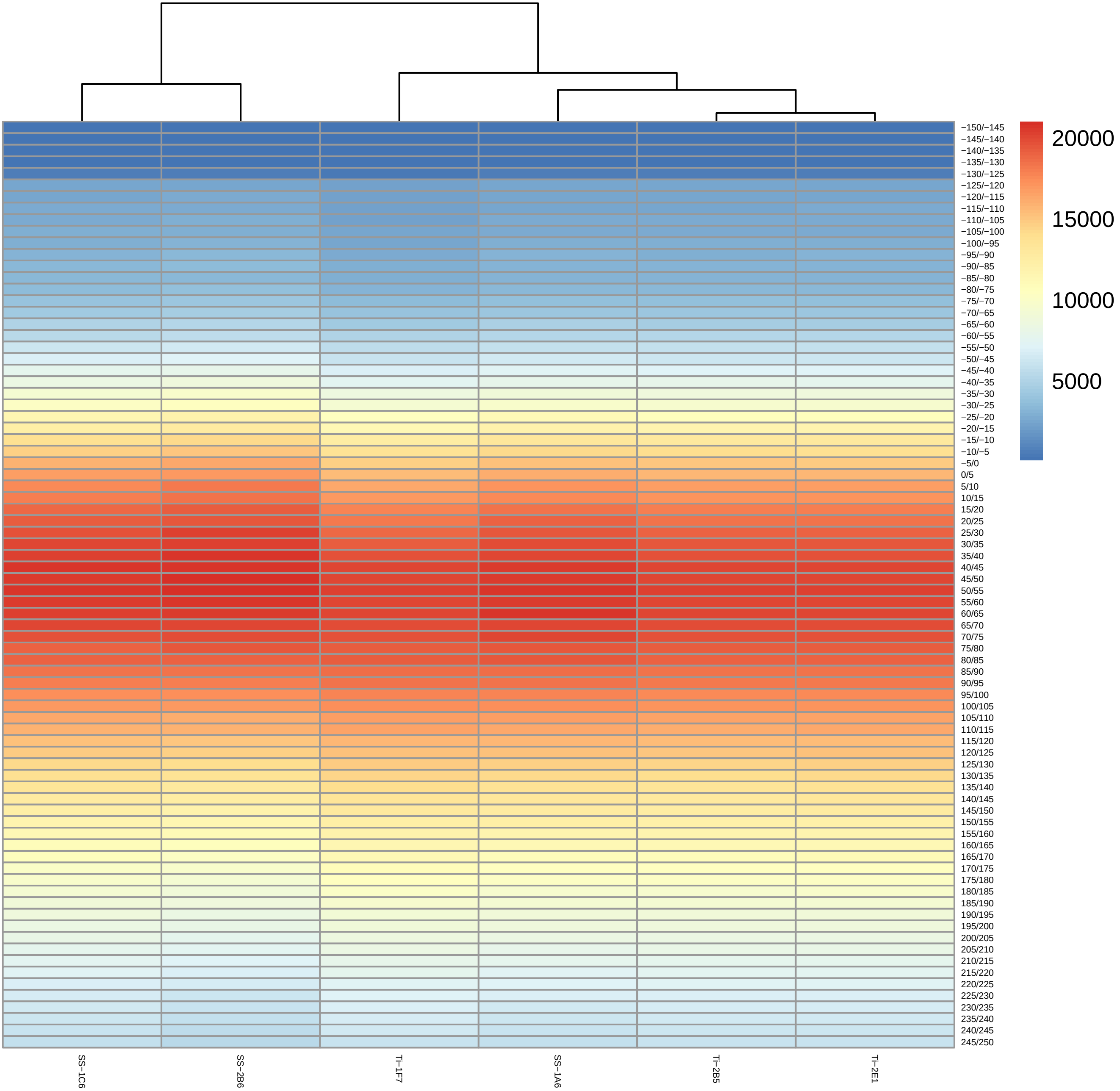

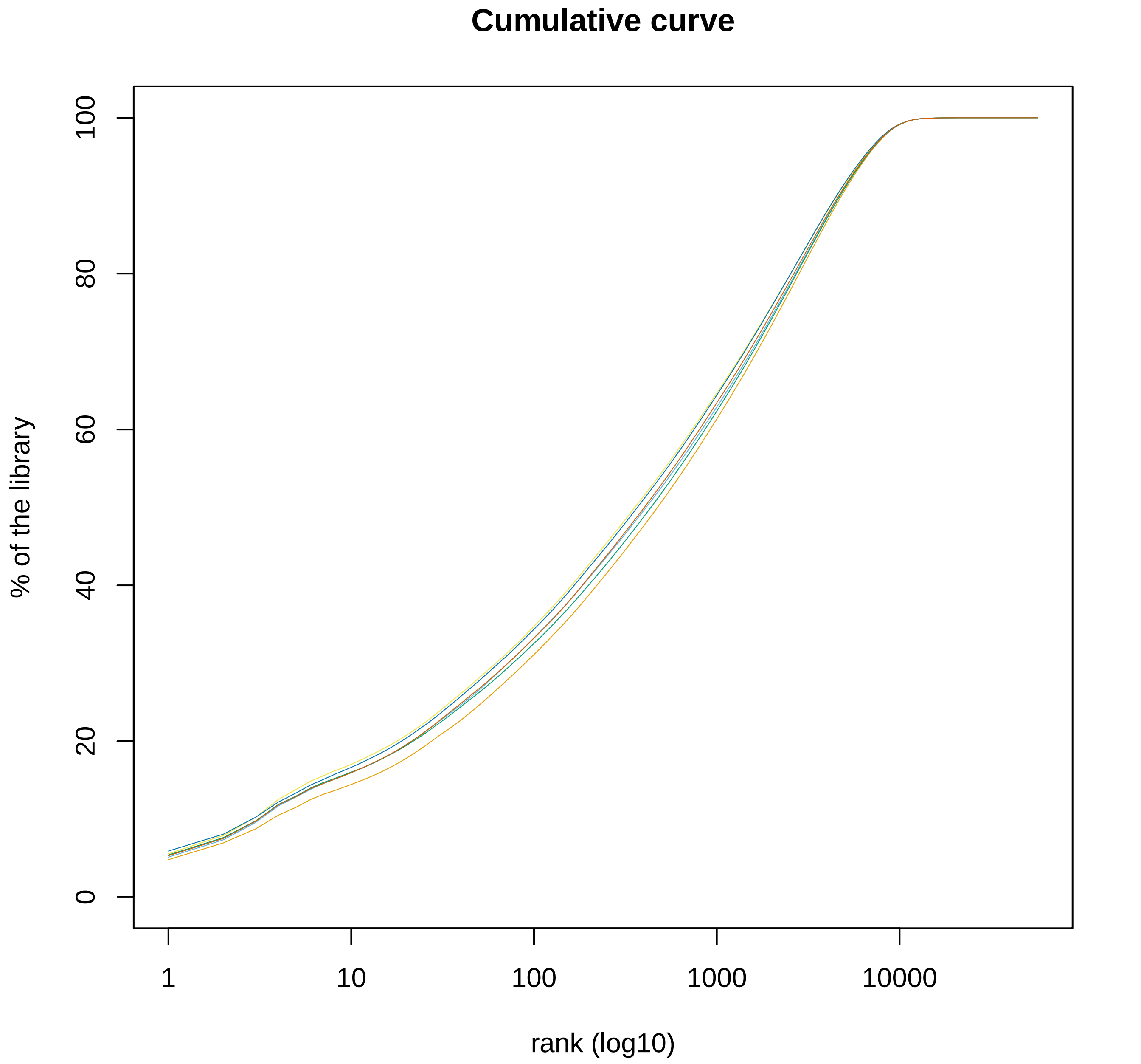
Cumulative curve
% of the library
0
20
40
60
80
100
1
10
100
1000
10000
rank (log10)

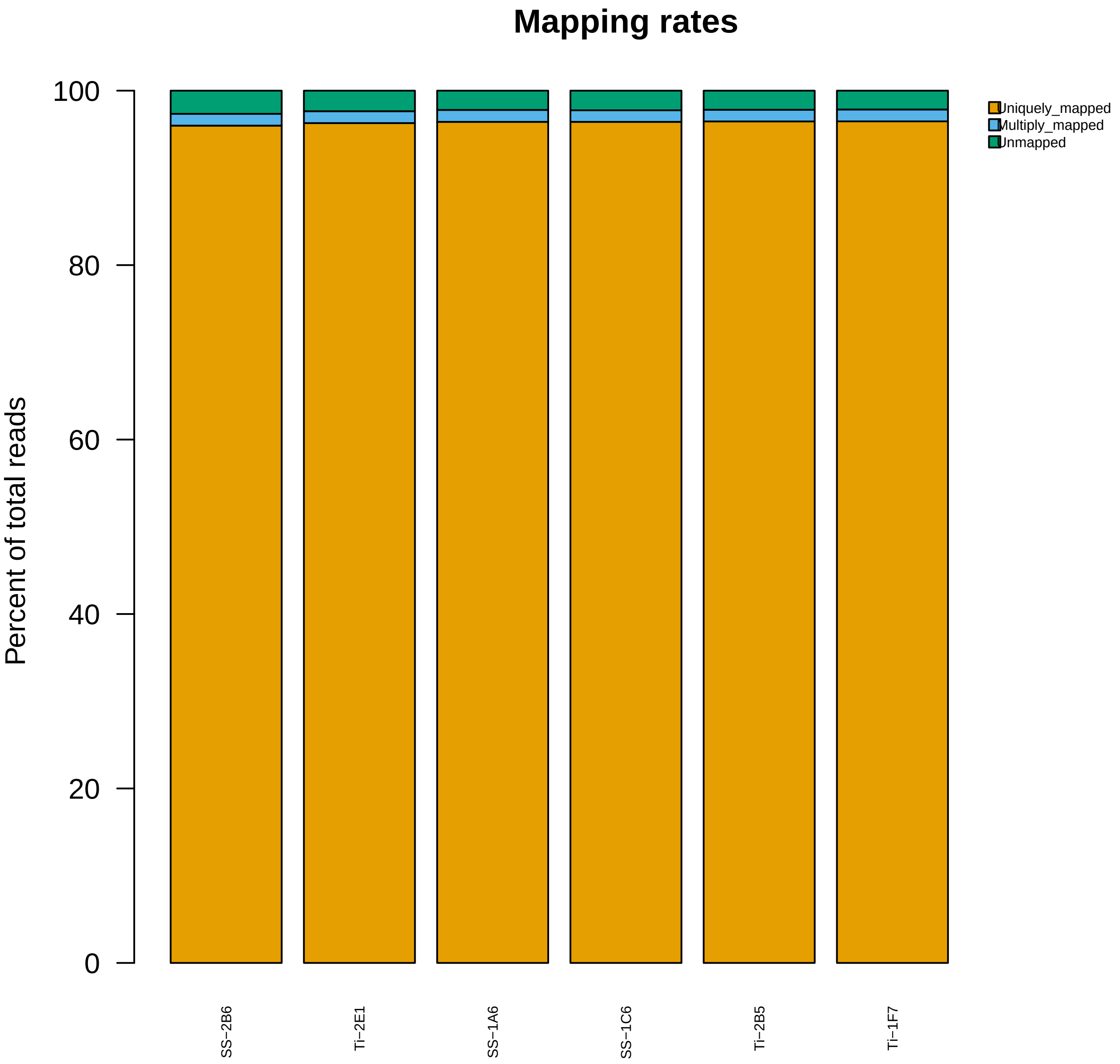

Mapping rates
Percent of total reads
0
20
40
60
80
100
Uniquely_mapped
Multiply_mapped
Unmapped
SS−2B6
Ti−2E1
SS−1A6
SS−1C6
Ti−2B5
Ti−1F7

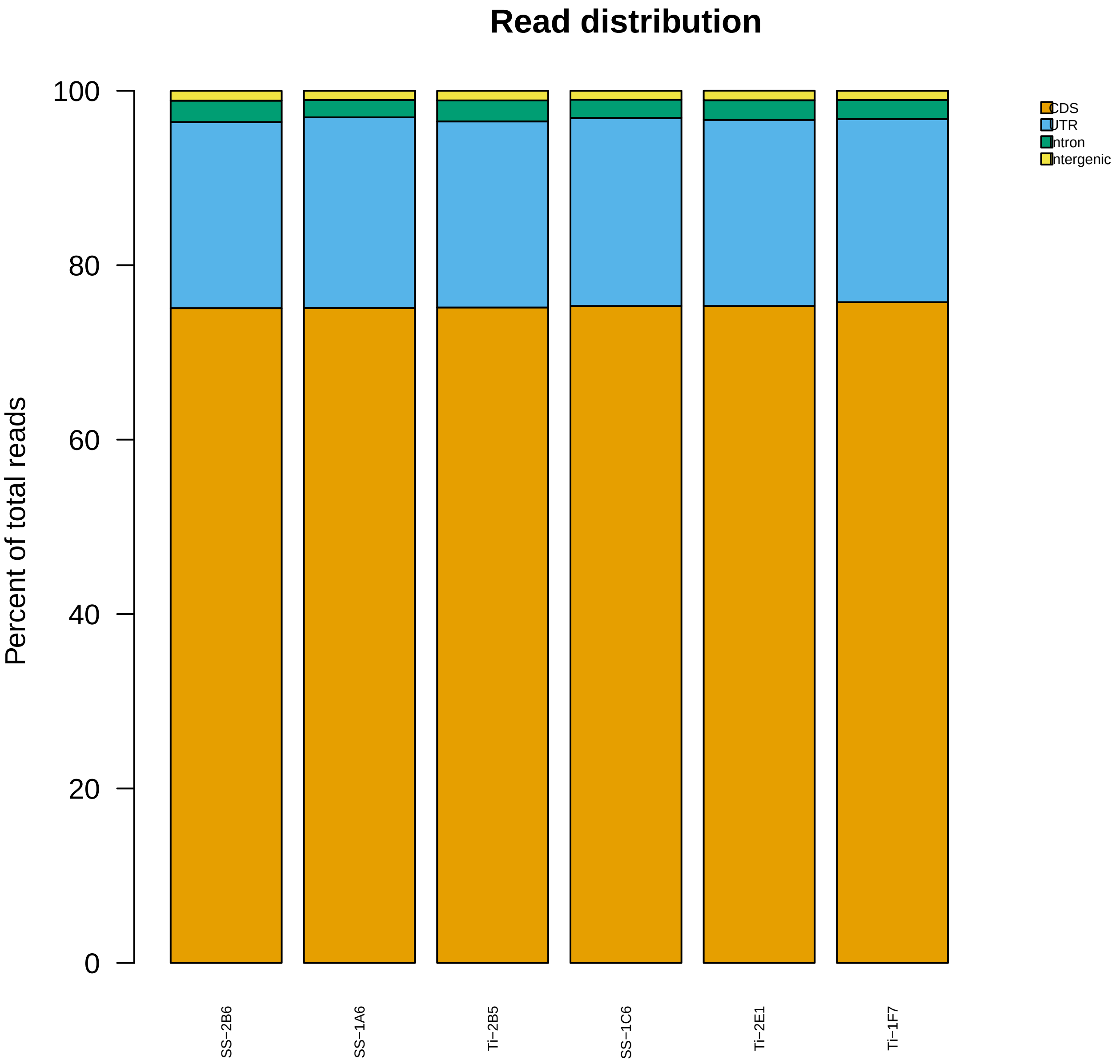

Read distribution
Percent of total reads
0
20
40
60
80
100
CDS
UTR
Intron
Intergenic
SS−2B6
SS−1A6
Ti−2B5
SS−1C6
Ti−2E1
Ti−1F7

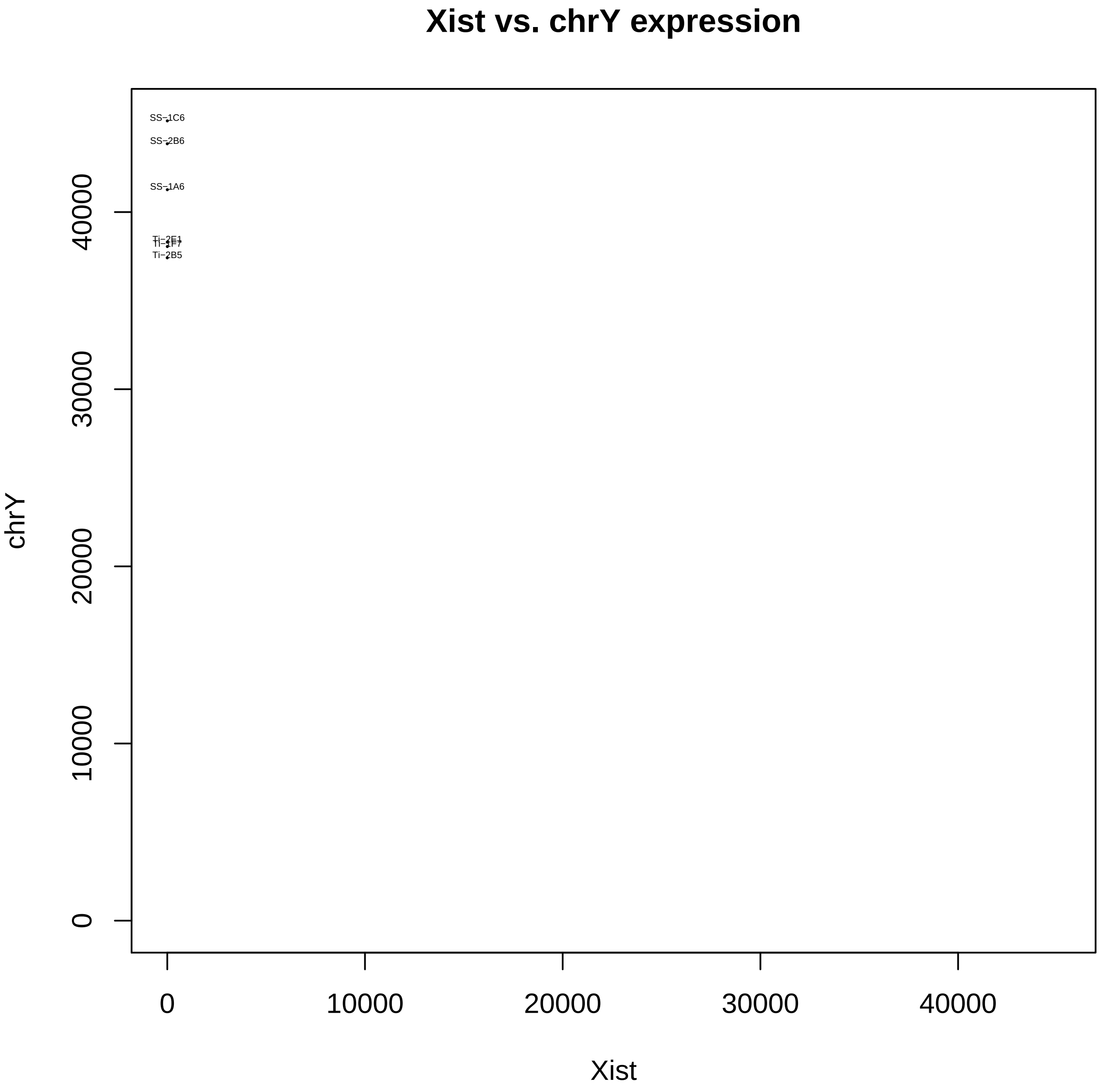
Xist vs. chrY expression
chrY
Xist
0
10000
20000
30000
40000
SS−1C6
SS−2B6
SS−1A6
Ti−2E1
Ti−1F7
Ti−2B5

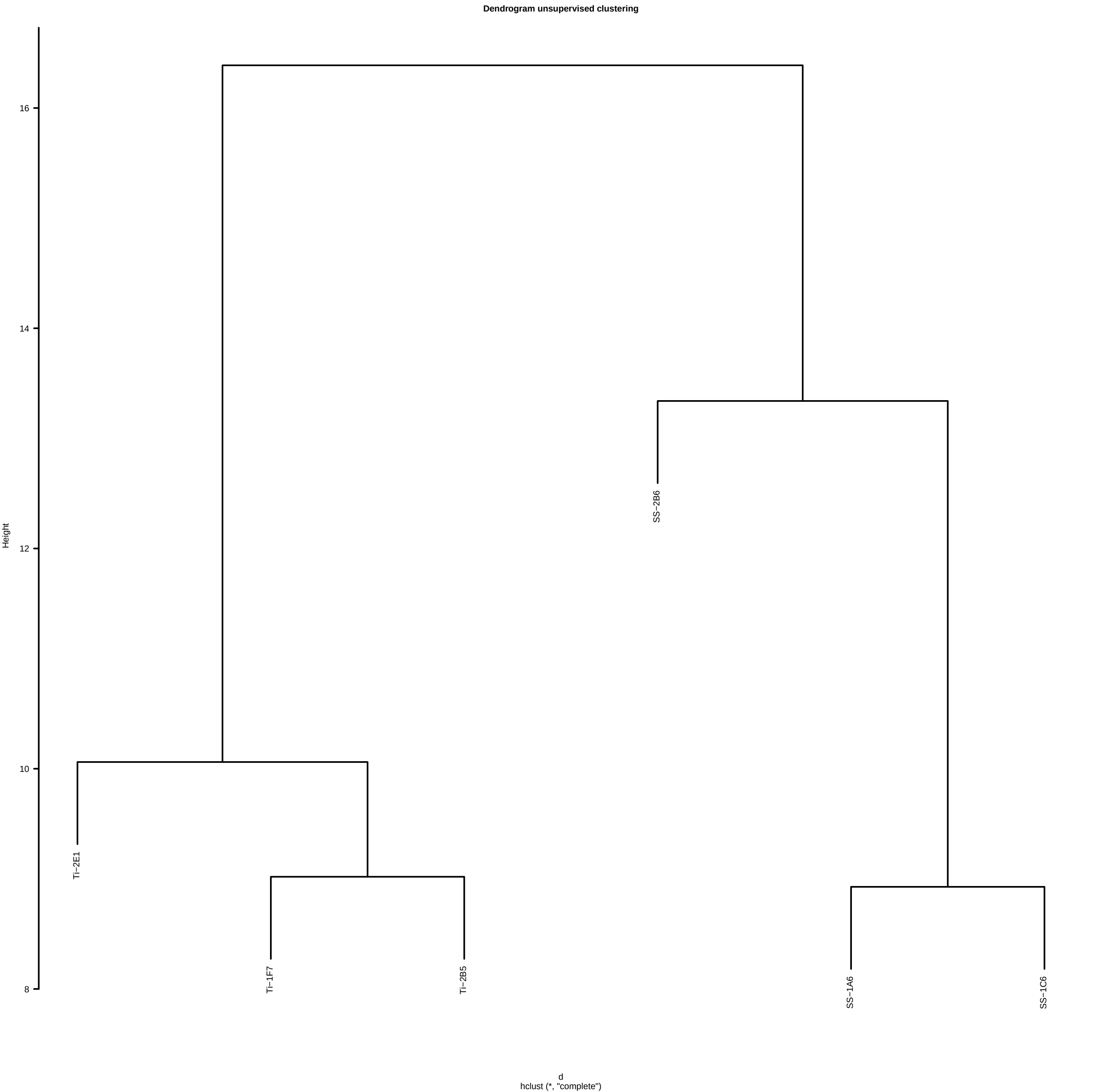
Dendrogram unsupervised clustering
Height
16
14
12
10
8
Ti−2E1
Ti−1F7
Ti−2B5
SS−2B6
SS−1A6
SS−1C6
d
hclust (*, "complete")

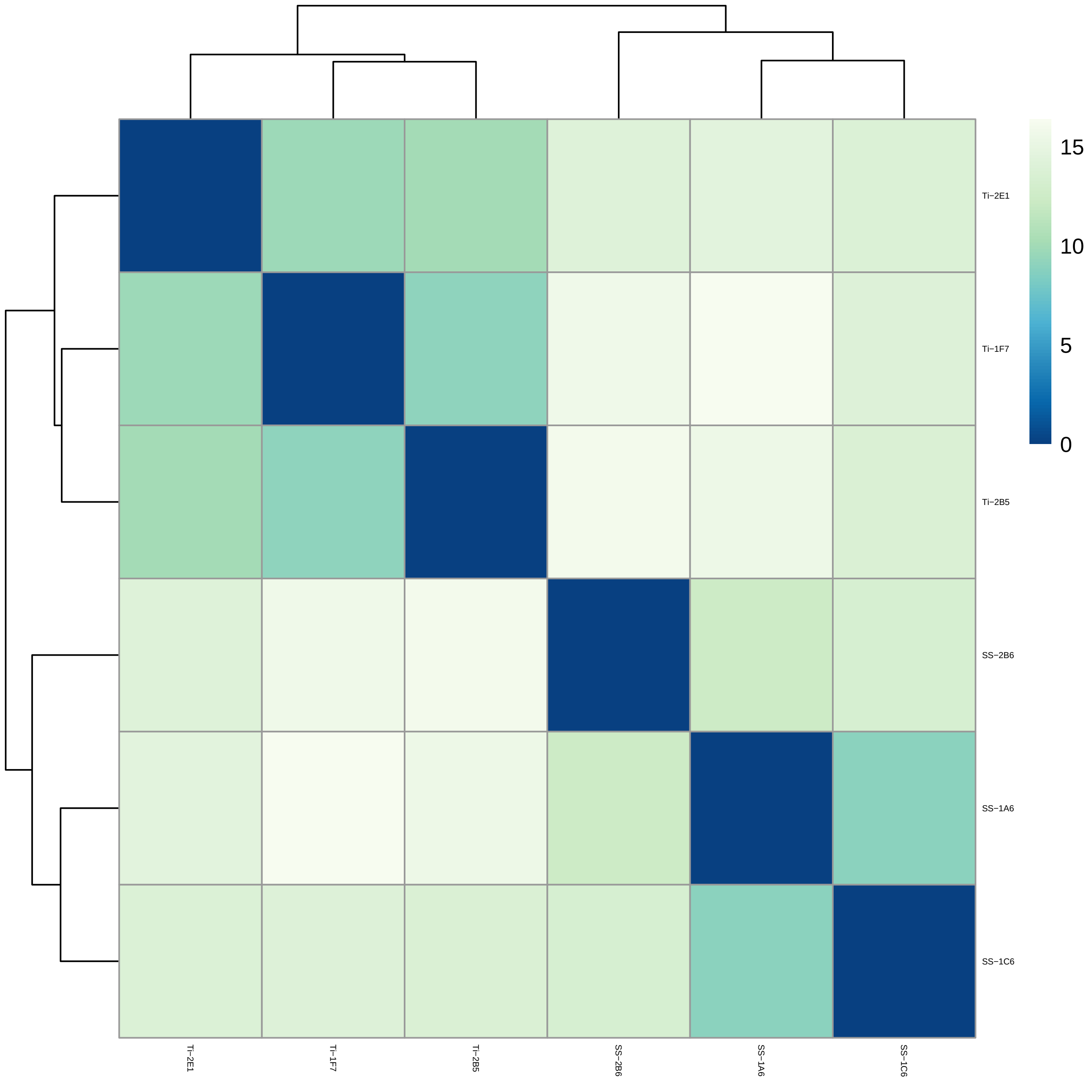

Ti−2E1
Ti−1F7
Ti−2B5
SS−2B6
SS−1A6
SS−1C6
15
10
5
0
Ti−2E1
Ti−1F7
Ti−2B5
SS−2B6
SS−1A6
SS−1C6

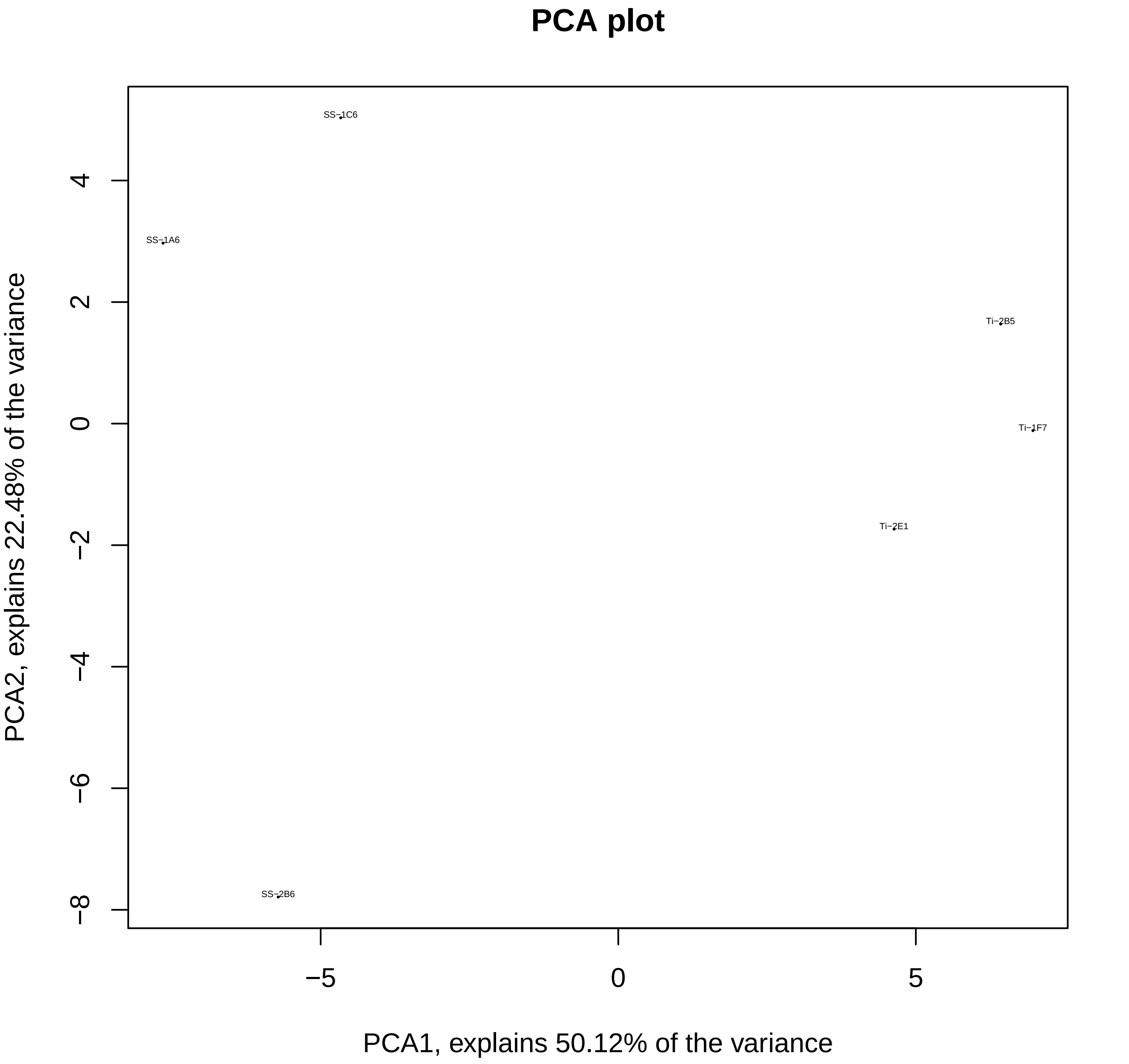
PCA plot
SS−1C6
SS−1A6
Ti−2B5
Ti−1F7
Ti−2E1
SS−2B6
4
2
0
−2
−4
−6
−8
−5
0
5
PCA1, explains 50.12% of the variance
PCA2, explains 22.48% of the variance

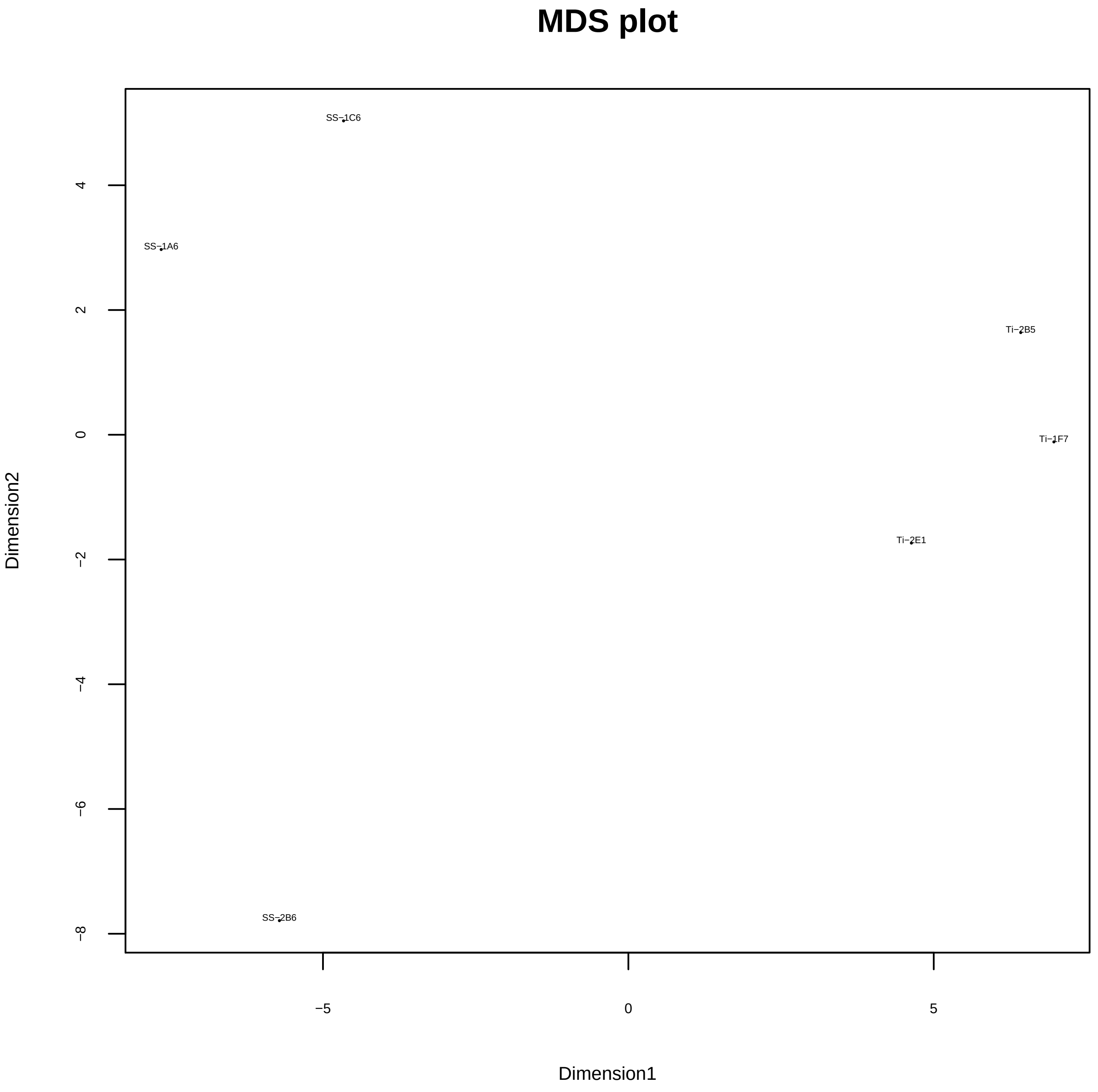
MDS plot
SS−1C6
SS−1A6
Ti−2B5
Ti−1F7
Ti−2E1
SS−2B6
4
2
0
−2
−4
−6
−8
−5
0
5
Dimension1
Dimension2
