# Supplementary material for: A biomimetic engineered bone platform for advanced testing of prosthetic implants
Source: Sci Rep. 2020 Dec 17;10:22154. doi: 10.1038/s41598-020-78416-w (PMC7747643; doi:10.1038/s41598-020-78416-w)
Supplement: Supplementary file 3 — Supplementary information. [file 41598_2020_78416_MOESM3_ESM.docx]

**A Biomimetic Engineered Human Bone Platform for Advanced Testing of Prosthetic Implants**

Martina Sladkova-Faurea^1,†^, Michael Pujari-Palmer^2,†^, Caroline Öhman-Mägi^2^, Alejandro López^2^, Hanbin Wang^1^, Håkan Engqvist^2^, Giuseppe Maria de Peppo^1,*^

^1^The New York Stem Cell Foundation Research Institute, New York, NY, USA.

^2^Division of Applied Materials Sciences, Uppsala University, Uppsala, Sweden.

^†^These authors contributed equally.

^*^Corresponding Author:

Giuseppe Maria de Peppo, PhD

NYSCF – Ralph Lauren Senior Research Investigator

The New York Stem Cell Foundation Research Institute, 619 West 54^th^ Street, New York, NY 10019, USA. Tel: +1-917-592-9923.

**
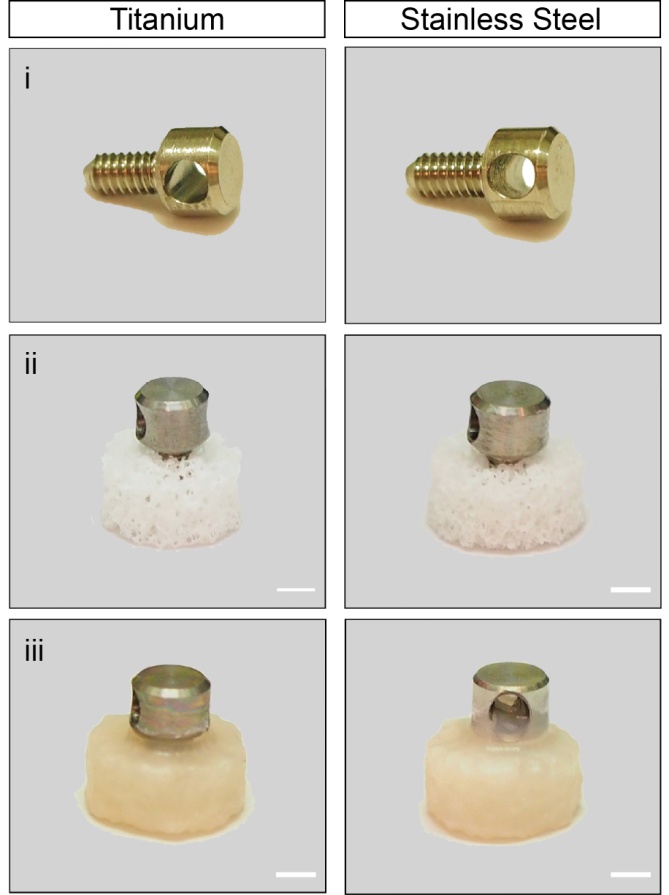
**

**Figure S1. Bone-implant platform.** Photographs of the titanium and stainless steel implants alone (i), the implants anchored into decellularized bone scaffolds, (ii) and the bone-implant platforms (iii) after 7 weeks of culture in an osteogenic environment. Scale bar: 2 mm.

**
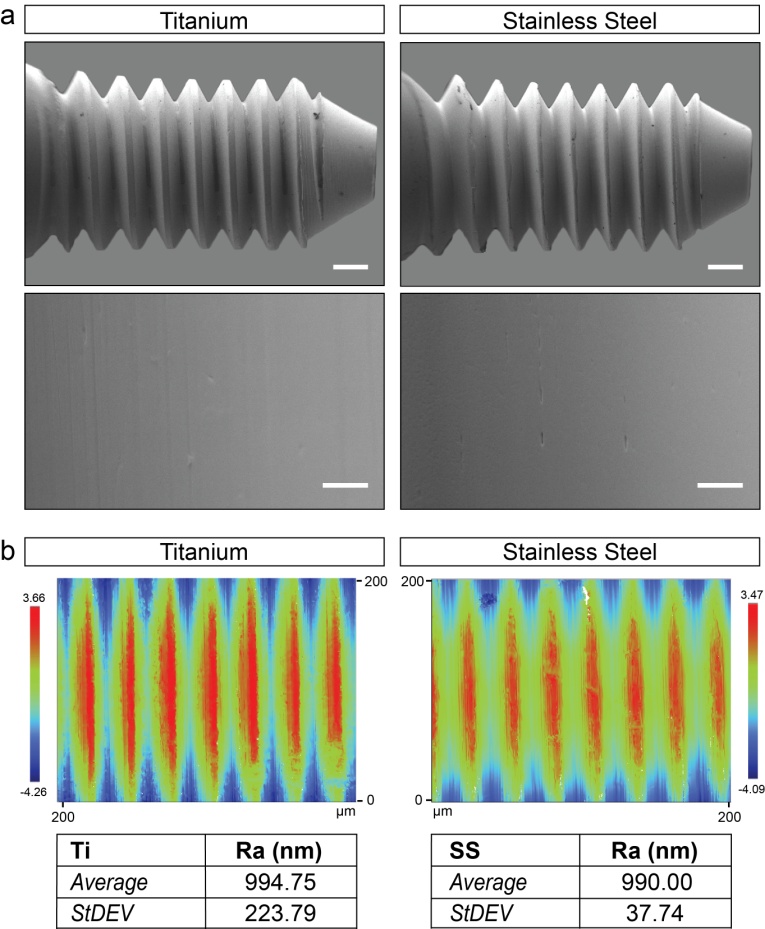
**

**Figure S2. Implants.** (a) Scanning electron micrographs of titanium and stainless steel model prosthetic implants. Scale bar: 500 µm (top) and 10 µm (bottom). (b) Optical profilometry images showing the surface topography of titanium and stainless steel implants with corresponding roughness values (Ra).

Abbreviations: Ra, arithmetical mean deviation of the profile; SS, stainless steel; Ti, titanium.

**Table S1.** **Density of decellularized bone scaffolds.**

**
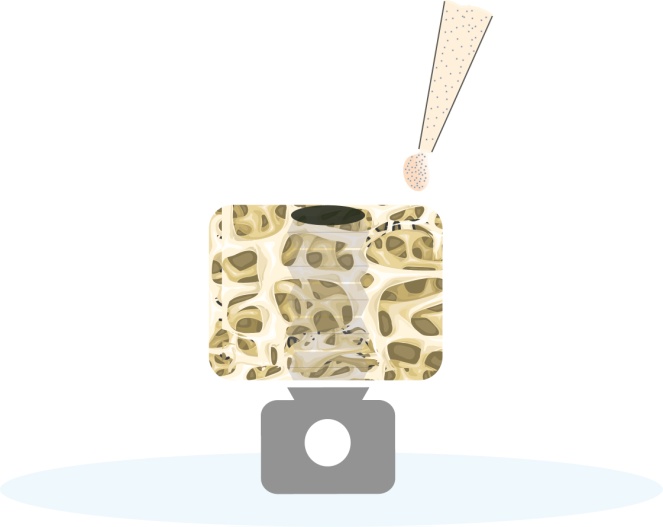
**

**Figure S3. Schematic of the cell seeding method.** The scaffold-implant constructs are placed upside down in 6-well plates, and the cells seeded onto the scaffolds using a pipette.

**
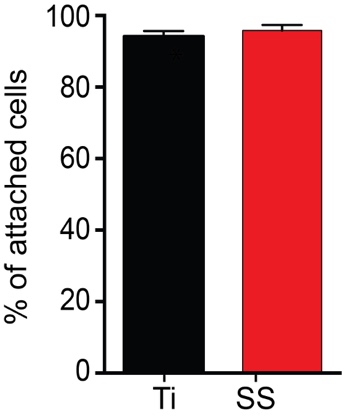
**

**Figure S4. Seeding efficiency.** Percentage of cells attached to the scaffolds anchoring the titanium and stainless steel implants. Data represent averages ± SD (n = 14, unpaired Student’s t-test).


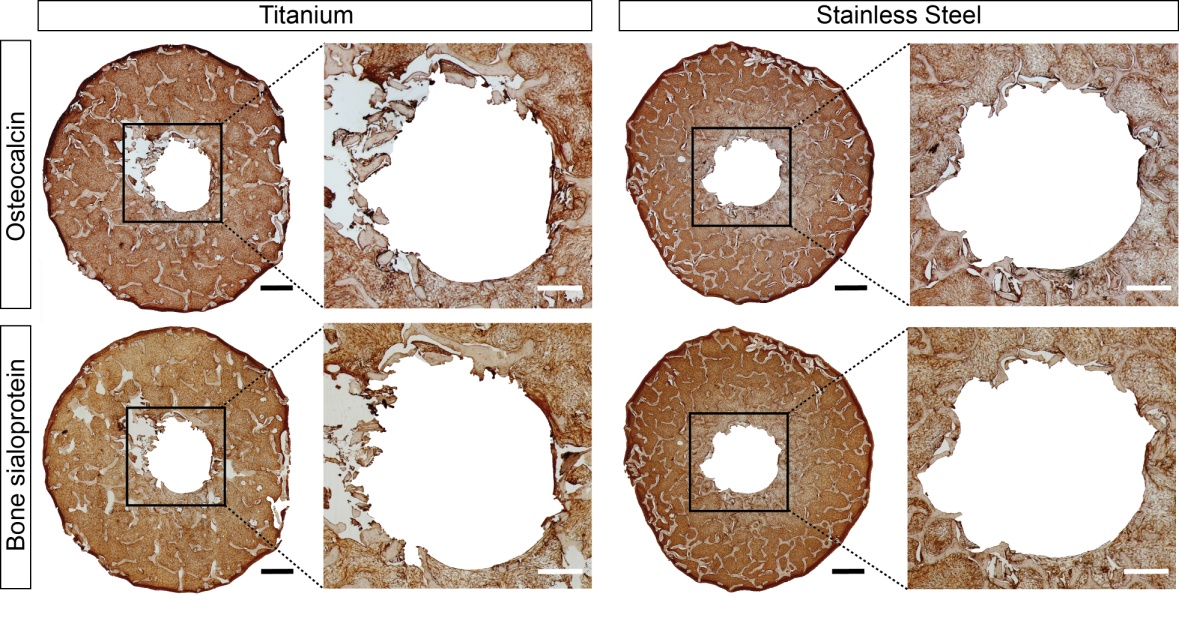


**Figure S5. Additional data on immunohistochemical staining.** Immunohistochemical analysis of samples after 7 weeks of culture in an osteogenic environment. Samples are positive (brown) for osteocalcin and bone sialoprotein. Scale bar: 1 mm (left) and 100 µm (right).

**
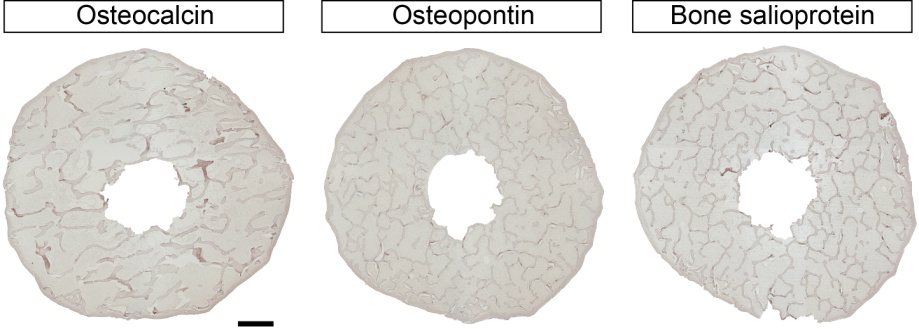
**

**Figure S6. Additional data on immunohistochemical staining.** Control samples staining negative for osteocalcin, osteopontin and bone sialoprotein. Samples were counterstained with hematoxylin. Images correspond to a sample stained omitting the secondary antibody (left), a sample stained omitting the primary antibody (middle), and a sample stained omitting the secondary antibody (right). Scale bar: 1 mm.

**Table S2. List of differentially expressed genes overexpressed in response to titanium implants.**

**Table S3. List of differentially expressed genes overexpressed in response to stainless steel implants.**

**Table S4. Ingenuity Pathway Analysis.**

**Table S5. Statistics.**

**
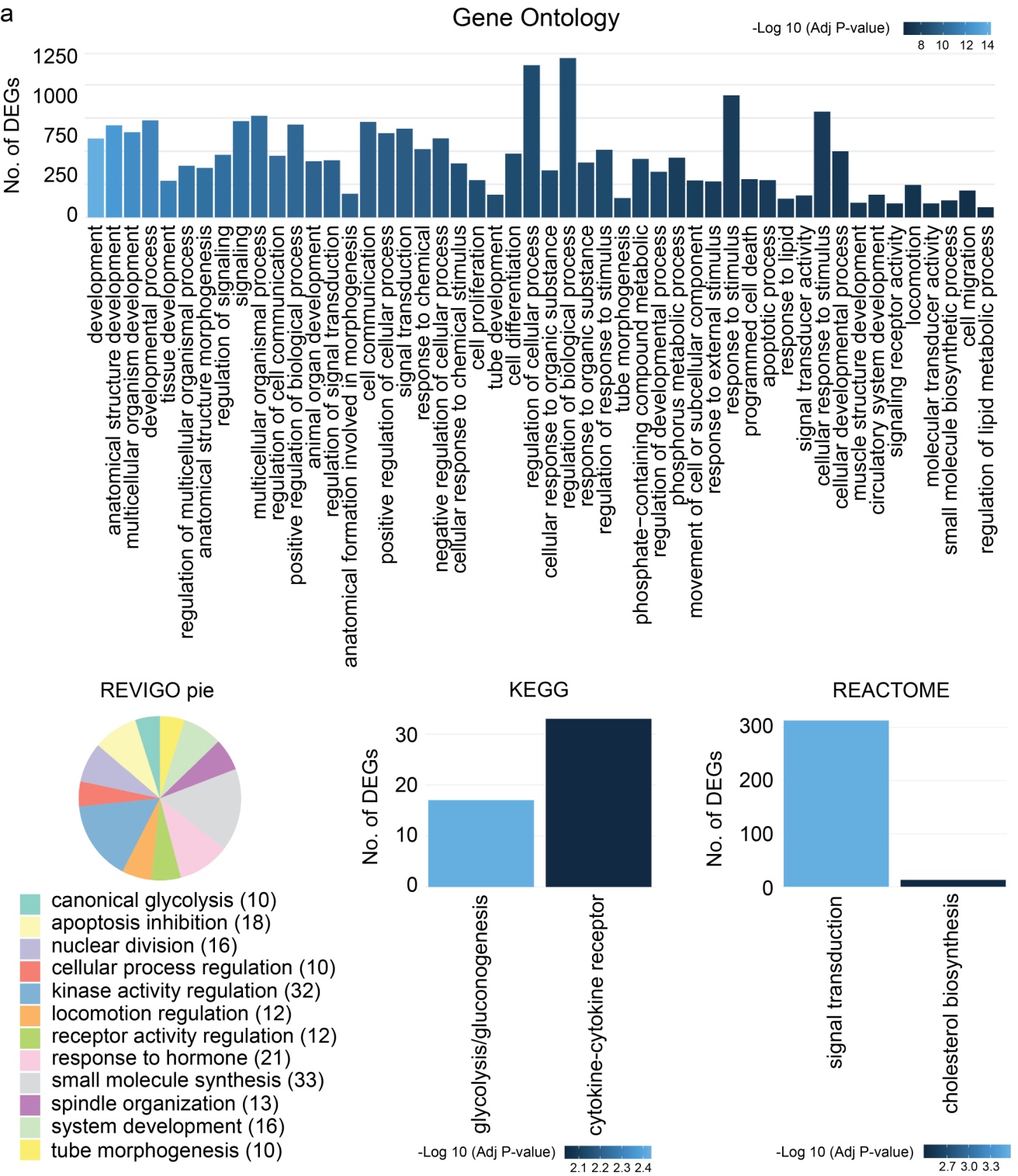
**

**
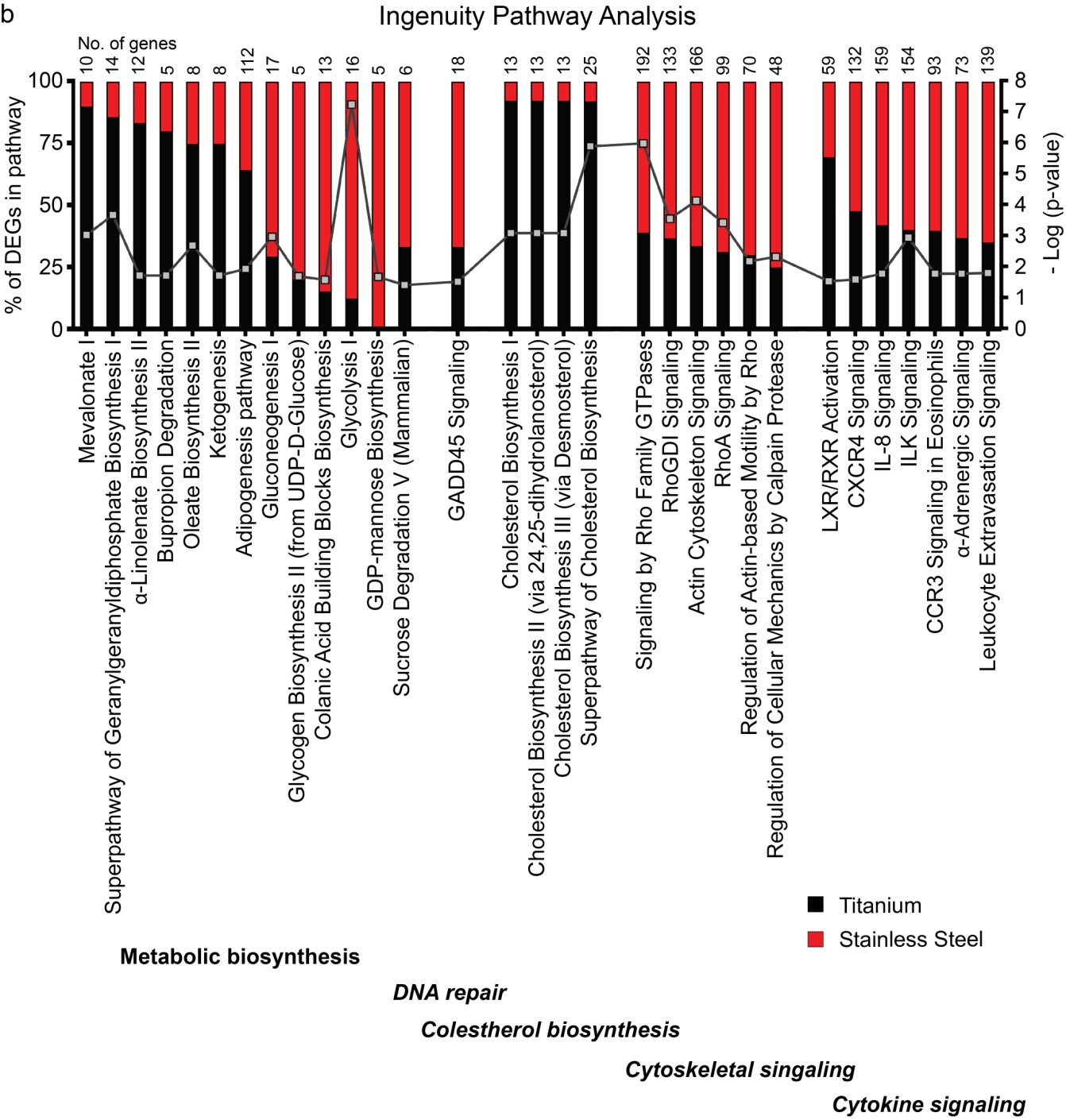
**

**Figure S7. Additional data on the molecular response to implants** (a) Functional classification of differentially expressed genes in response to titanium and stainless steel implants using the gene ontology (GO) database, REVIGO, and the KEGG and Reactome Pathway database. (b) Ingenuity Pathway Analysis of differentially expressed genes showing important signaling pathways regulated in response to titanium and stainless steel implants. Above each bar the number of DEGs falling in each category.

Abbreviations: DEGs, differentially expressed genes.

**
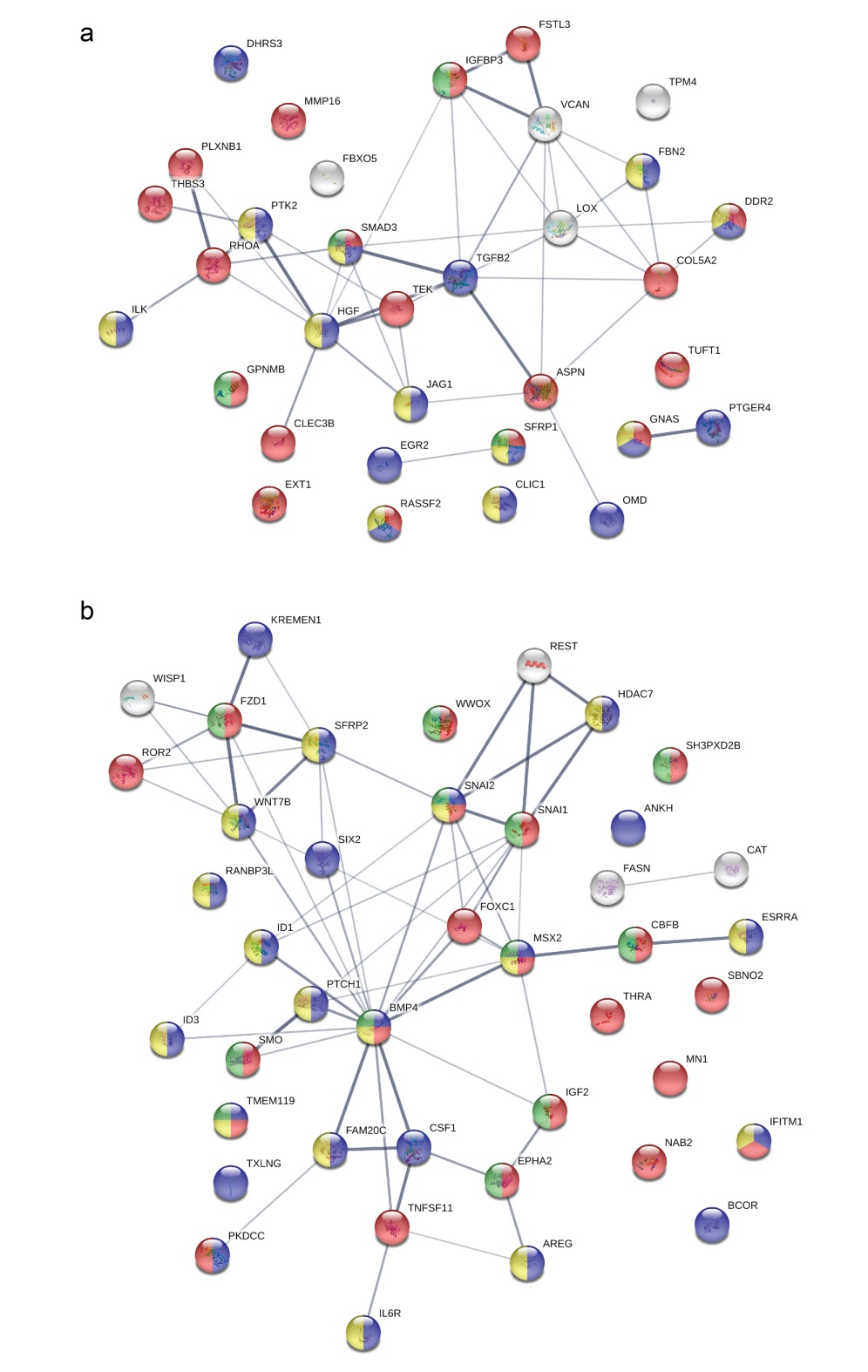
**

**Figure S8. Ossification gene network.** Interaction network of genes involved in the ossification process over-expressed in response to titanium (a) and stainless steel (b) implants.

Node colors indicate association with the gene ontology (GO) terms ossification (blue), regulation of ossification (red), osteoblast differentiation (green), and regulation of osteoblast differentiation (yellow). The color saturation of the edges represents the confidence score of a functional association.


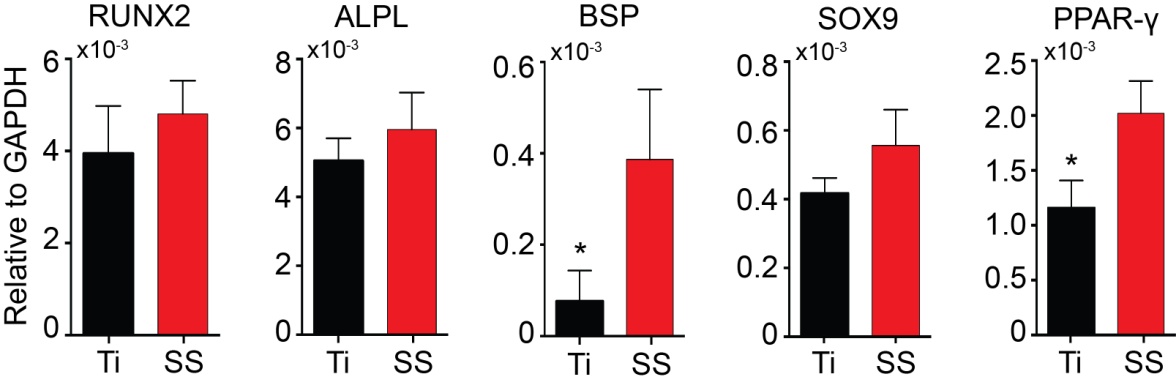


**Figure S9. Expression of mesodermal genes.** Real-time PCR data showing the expression of runt-related transcription factor 2 (RUNX2), liver/bone/kidney alkaline phosphatase (ALPL), integrin binding sialoprotein (IBSP), sex-determining region Y-box 9 (SOX-9), and peroxisome proliferator-activated receptor gamma (PPAR-γ) after 7 weeks of culture in response to titanium and stainless steel implants. Data represent averages ± SD (n = 3, unpaired Student’s t-test; asterisk denotes significant difference between the titanium and stainless steel groups).

**
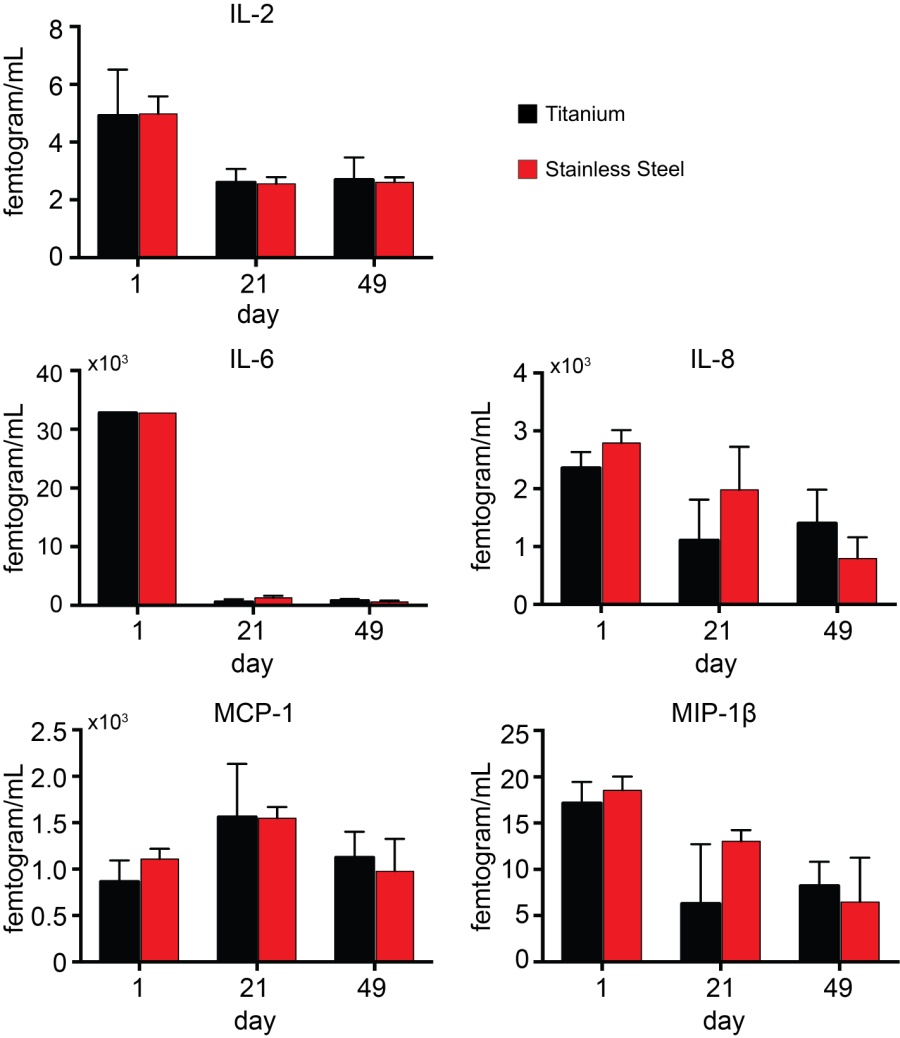
**

**Figure S10. Release of trophic factors.** Release of interleukin 2 (IL-2), interleukin 6 (IL-6), interleukin 8 (IL-8), monocyte chemoattractant protein 1 (MCP-1), and macrophage inflammatory protein 1 beta (MIP-1β) 1 day, 3 weeks and 5 weeks after culture in response to titanium and stainless steel implants. Data represent averages ± SD (n = 3, unpaired Student’s t-test).

**
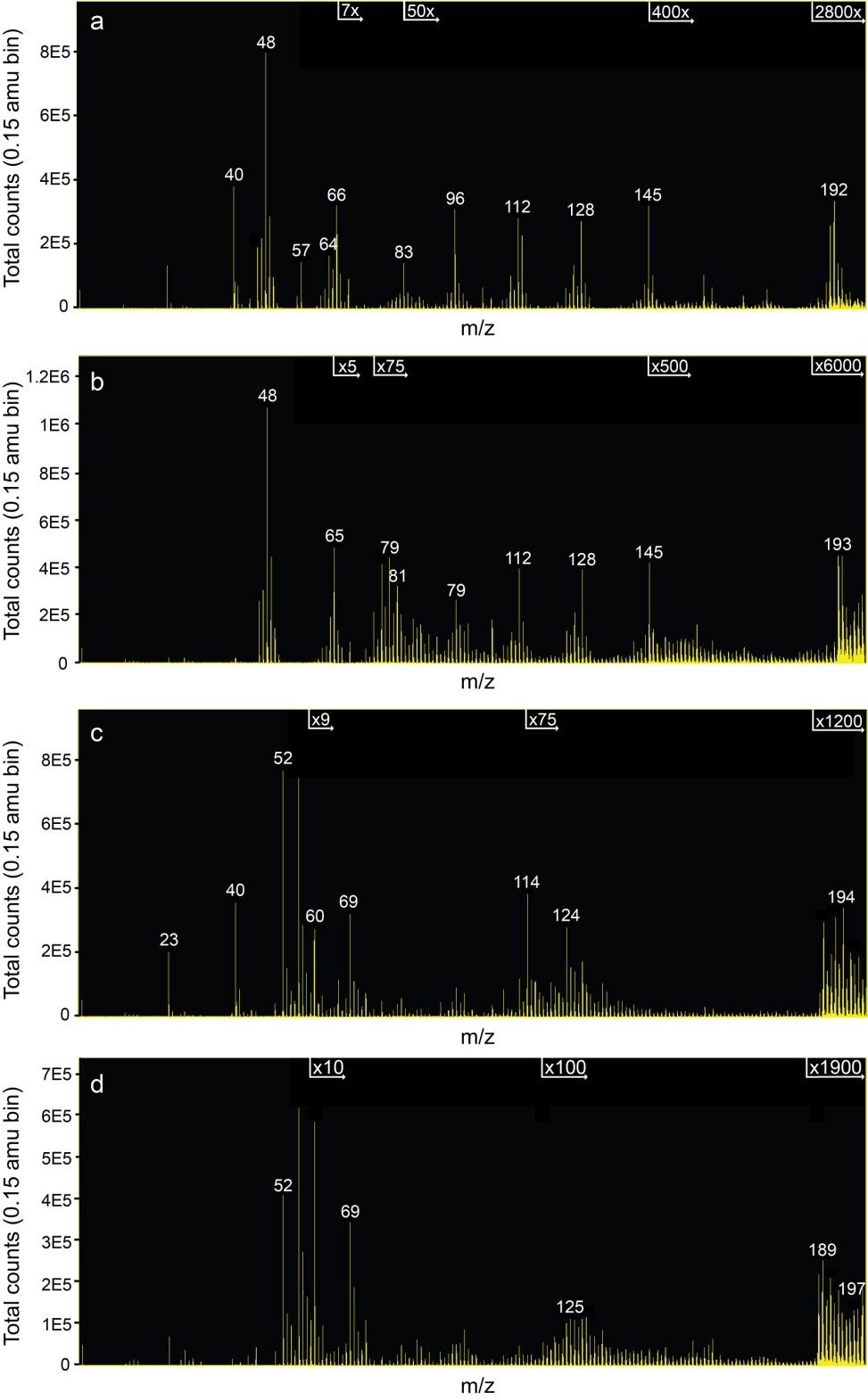
**

**Figure S11. Additional data on Tof-SIMS analysis.** Representative Tof-SIMS view of titanium (a and b) and stainless steel (c and d) samples after 7 weeks of culture in an osteogenic environment recorded over analysis areas of 600 x 600 µm^2^ (a and c) and 200 x 200 µm^2^ (b and d).

Abbreviations: m, mass; z, charge.

**
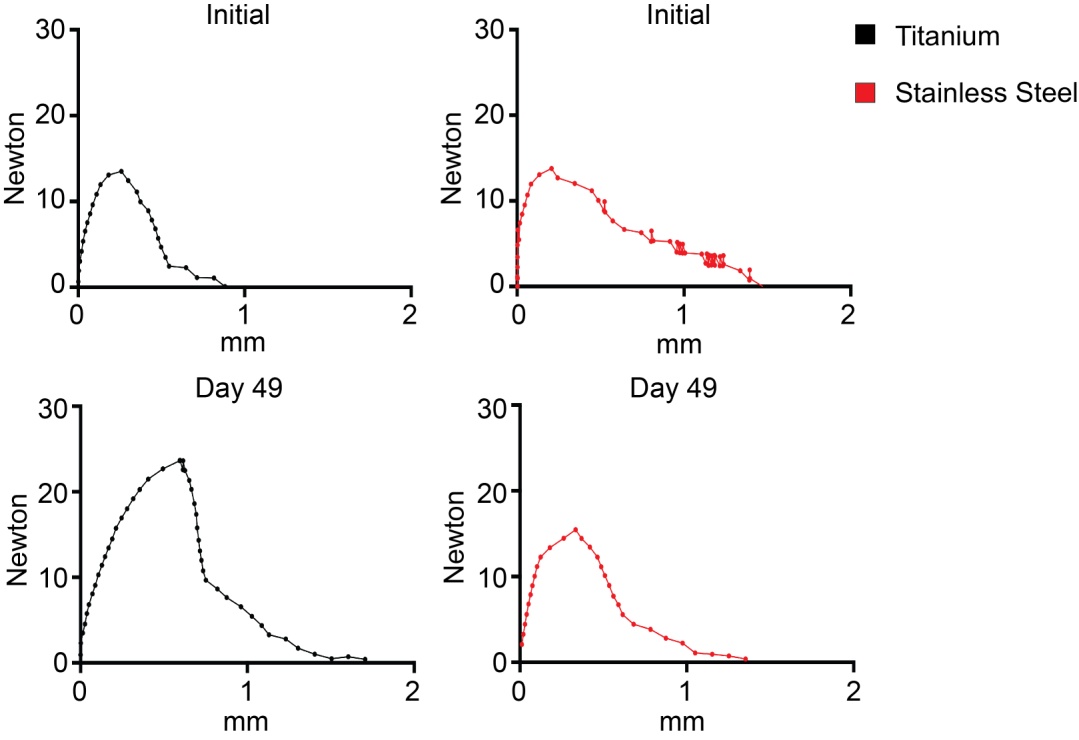
**

**Figure S12. Stress-displacement curves.** Representative plots showing the stress-displacement curves for titanium and stainless steel implants anchored to decellularized bone scaffolds (initial) and tissue-engineered human bone after 49 days of culture in an osteogenic environment.

**Video S1. Pullout test.**

**
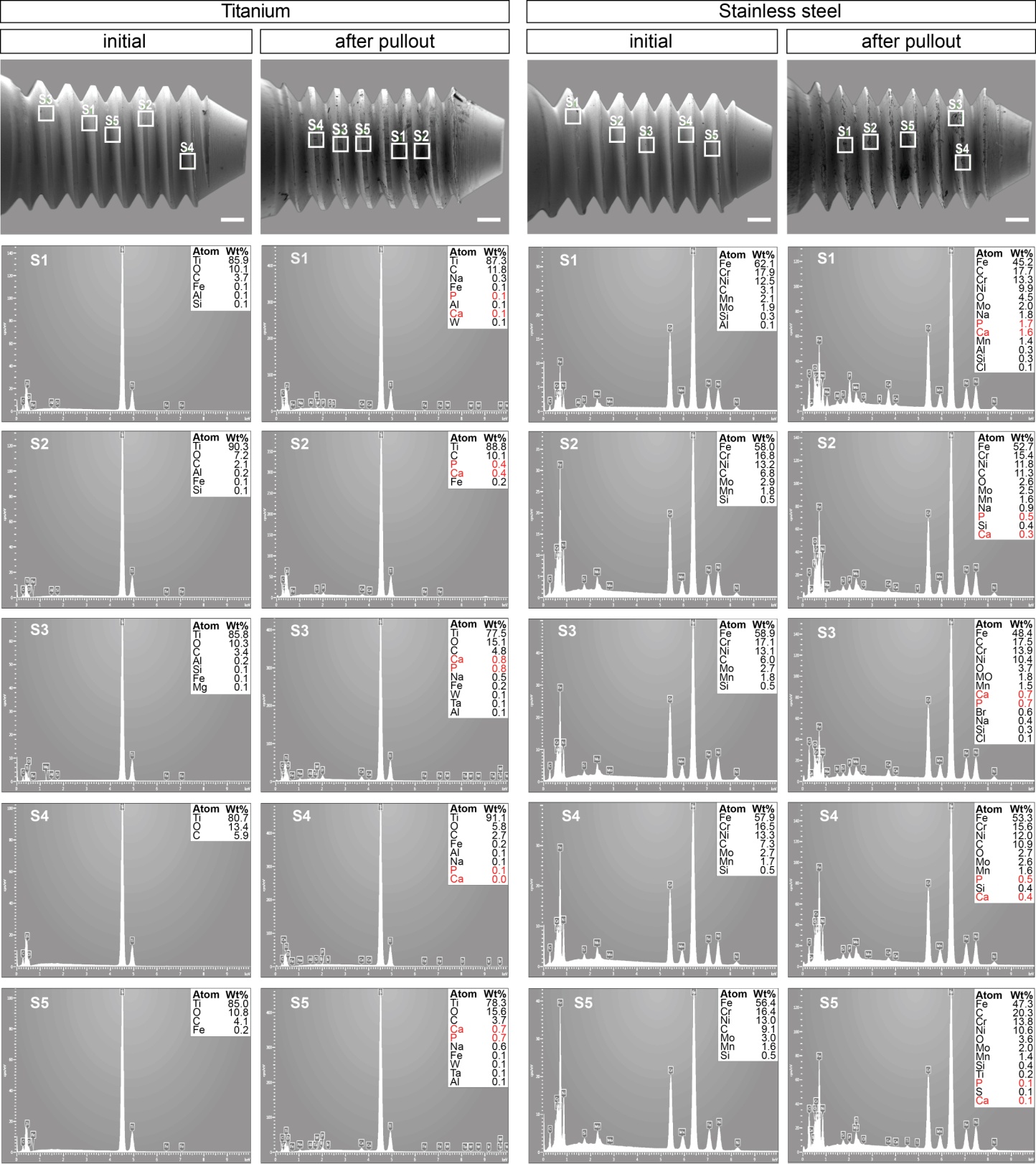
**

**Figure S13. Elemental mapping of implants.** Energy-dispersive X-ray spectroscopy characterization of titanium and stainless steel implants before insertion and following pullout after 7 weeks of culture in an osteogenic environment.

Abbreviations: Wt, weight; S, site.
